# Genome-wide analysis of regulatory proteases sequences identified through bioinformatics data mining in *Taenia solium*

**DOI:** 10.1186/1471-2164-15-428

**Published:** 2014-06-04

**Authors:** Hong-Bin Yan, Zhong-Zi Lou, Li Li, Paul J Brindley, Yadong Zheng, Xuenong Luo, Junling Hou, Aijiang Guo, Wan-Zhong Jia, Xuepeng Cai

**Affiliations:** State Key Laboratory of Veterinary Etiological Biology, Key Laboratory of Veterinary Parasitology of Gansu Province, Key Laboratory of Veterinary Public Health of Agriculture Ministry, Lanzhou Veterinary Research Institute, Chinese Academy of Agricultural Sciences, Lanzhou, 730046 Gansu Province PR China; Department of Microbiology, Immunology & Tropical Medicine, and Research Center for Neglected Diseases of Poverty, School of Medicine & Health Sciences, The George Washington University, Washington, DC USA

**Keywords:** Proteases, *Taenia solium*, Drug target, Vaccine candidate antigen, Genome-wide analysis, Cysticercosis, Platyhelminth

## Abstract

**Background:**

Cysticercosis remains a major neglected tropical disease of humanity in many regions, especially in sub-Saharan Africa, Central America and elsewhere. Owing to the emerging drug resistance and the inability of current drugs to prevent re-infection, identification of novel vaccines and chemotherapeutic agents against *Taenia solium* and related helminth pathogens is a public health priority. The *T. solium* genome and the predicted proteome were reported recently, providing a wealth of information from which new interventional targets might be identified. In order to characterize and classify the entire repertoire of protease-encoding genes of *T. solium*, which act fundamental biological roles in all life processes, we analyzed the predicted proteins of this cestode through a combination of bioinformatics tools. Functional annotation was performed to yield insights into the signaling processes relevant to the complex developmental cycle of this tapeworm and to highlight a suite of the proteases as potential intervention targets.

**Results:**

Within the genome of this helminth parasite, we identified 200 open reading frames encoding proteases from five clans, which correspond to 1.68% of the 11,902 protein-encoding genes predicted to be present in its genome. These proteases include calpains, cytosolic, mitochondrial signal peptidases, ubiquitylation related proteins, and others. Many not only show significant similarity to proteases in the Conserved Domain Database but have conserved active sites and catalytic domains. KEGG Automatic Annotation Server (KAAS) analysis indicated that ~60% of these proteases share strong sequence identities with proteins of the KEGG database, which are involved in human disease, metabolic pathways, genetic information processes, cellular processes, environmental information processes and organismal systems. Also, we identified signal peptides and transmembrane helices through comparative analysis with classes of important regulatory proteases. Phylogenetic analysis using Bayes approach provided support for inferring functional divergence among regulatory cysteine and serine proteases.

**Conclusion:**

Numerous putative proteases were identified for the first time in *T. solium*, and important regulatory proteases have been predicted. This comprehensive analysis not only complements the growing knowledge base of proteolytic enzymes, but also provides a platform from which to expand knowledge of cestode proteases and to explore their biochemistry and potential as intervention targets.

**Electronic supplementary material:**

The online version of this article (doi: 10.1186/1471-2164-15-428) contains supplementary material, which is available to authorized users.

## Background

Taeniosis and cysticercosis caused by adult and larval stages of the *Taenia solium* (Platyhelminthes: Cestoda, Cyclophyllidea, Taeniidae) parasite, respectively, remain important parasitic diseases and a major health and economic burdens in less developed countries. Moreover, these infectious diseases also are increasingly seen in more developed countries because of immigration from endemic areas where pigs are reared and pork is consumed [1]. *T. solium* has a complex, two-host developmental cycle. Humans are the only definitive host - harboring the adult tapeworm, which result in taeniasis, whereas pigs, humans and other mammals can serve as intermediate hosts for the larval (cysticercus) form [2, 3]. Infection of the human nervous system by the cysticercus leads to neurocysticercosis with the symptoms of acquired epilepsy and seizure.

Therapeutic measures available to treat neurocysticercosis include steroids, treatments for symptoms, surgery, and antiparasitic drugs to kill cysticerci in the central nervous system, muscles, and other sites. By contrast, infection of the human small intestine by the adult developmental stage of tapeworm is usually asymptomatic, and readily treated with the oral medication praziquantel. However, attempts to date to control transmission of the parasite have often been poorly effective and not sustainable [1–3].

Over the past decade, research has been undertaken to develop vaccines and novel chemotherapeutic agents for use in pigs to prevent and control transmission of *T. solium*. Whereas noteworthy progress has been made [3–6], no ideal vaccine is currently available for immunization to prevent infection of porcine or human populations at risk of infection. The screening and identification of ideal surface receptors or other proteins as molecular targets is the key step for the development of effective prevention and control strategy. Since the oncosphere stage that is released from the egg in the small intestine of the pig (or human) and which is then activated by the action of intestinal enzymes and bile salts is the first stage determining the infection success in their intermediate host, the identification of oncospheral antigens represents a key step to clarify their specific roles in the biology of the parasite–host relationship. In recent years, an antigen termed TSOL18 has proven to be effective as an immunogen in trials and naturally acquired infection with *T. solium* in pigs [4–6]. No new vaccines or drugs against cysticercosis, however, have been registered in recent years [1]. Accordingly, it is sensible to explore and seek novel molecular targets and their potential for vaccines and chemotherapeutic agents to block transmission of this cestode.

Proteases have been examined in depth in immunological or chemotherapeutic studies aiming to develop anti-protozoa agents; this focus relates to the critical roles proteolytic enzymes play in the developmental cycles of the parasites [7]. Moreover, proteases are important regulatory elements in all cells [8, 9]. They also play a key role as effectors of virulence in pathogens through converting host signal transduction and modifying the immune response [10–13]. However, few proteolytic enzymes have been identified or characterized for functions and interactions in *T. solium* and other cestodes (Cestoda).

Although five main catalytic classes of proteases have been identified from activated oncospheres in vitro of *T. solium* by proteomic analysis, only several have been described in depth [14]. Three proteases of *T. solium* have been named and classified in MEROPS database; among them, a cDNA encoding TsCL-1 - cathepsin L-like cysteine protease from the *T. solium* metacestode has been identified and the biochemical properties of the recombinant enzyme characterized [15]. Few proteases have been investigated for the potential to serve as chemotherapeutic targets or vaccine candidates against cysticercosis [15–20].

The newly available genome sequences of *T. solium* provide new avenues to discover novel vaccine candidates or therapeutic targets [21]. These abundant new data combined with specialized databases and bioinformatics techniques should accelerate the identification of anti-cestode agents, not the least by supplementing current proteomic identification techniques [22]. With this backdrop, here we investigated the *T. solium* genome for protease genes to provide first foundation of characterizing some potential targets. We identified numerous proteases in this cestode, many of which may have critical functions and hence be targeted with novel interventions.

## Results and discussion

Together, 200 predicted proteases belonging to 37 families were identified, excluding the inactive homologs or pseudogenes (Table 1; Additional file 1). The proteases constitute 1.68% of the 11,902 predicted protein-encoding genes of *T. solium*. There are currently three known or putative proteases identified in *T. solium* in the MEROPS database [15]. Proteases of five classes were characterized: 12%, 25%, 34.5%, 20.5%, and 8% for aspartic, cysteine, metallo-, serine, and threonine proteases, respectively. These proportions are consistent with other organisms [12, 23]. This study provided an exponential expansion in numbers of putative proteases from *T. solium*: more than 98% of the genes reported here are new.

**Table 1:** **Overview of characteristics of putative protease sequences encoded by the genome of** ***Taenia solium***

Protease class	Numbers of sequences	Numbers of families	Proteases with predicted transmembrane helices		Proteases with signal sequence
			TMMOD	TMHMM	
Aspartic	24	2	2	2	3
Cysteine	50	9	3	6	5
Metallo	69	16	17	19	11
Serine	41	8	18	19	13
Threonine	16	2	0	0	2
**Totals**	**200**	**37**	**40**	**46**	**34**

The genome of the human blood fluke *Schistosoma mansoni* and the model nematode *Caenorhabditis elegans* are well characterized and annotated; there are 196 *S. mansoni* and 369 *C. elegans* known or putative proteases in the MEROPS database. Moreover, a recent in depth critical analysis indicated the presence of at least 255 proteases in *S. mansoni*[12]. While the proteases proportions of each class are approximately equal, we observed that an obvious expansion in the relative proportion of aspartic proteases in *T. solium* compared to *S. mansoni* and *C. elegans*, a modest expansion of threonine proteases and a slight reduction of serine proteases in *T. solium* (Table 2). In general, these kinds of differences may result from evolutionary divergence, ecology, developmental life cycles and other aspects among species; e.g. *C. elegans* is a free living nematodes, *S. mansoni* is an obligate parasite of humans, and *T. solium* is a cyclophyllidean cestode. Differences among these three species, however, may also partially be due to the coverage and sequence quality of the genomes.

**Table 2:** **Proportions of protease families in the genomes of** ***Taenia solium*** **,** ***Schistosoma mansoni*** **and** ***Caenorhabditis elegans***

Protease class	***T. solium*** (%)	***S. mansoni*** (%)	***C. elegans*** (%)
Aspartic	12	4	5
Cysteine	25	27	20
Metallo	34.5	39	41
Serine	20.5	24	29
Threonine	8	6	5
**Totals**	**100**	**100**	**100**

**Figure 1:**
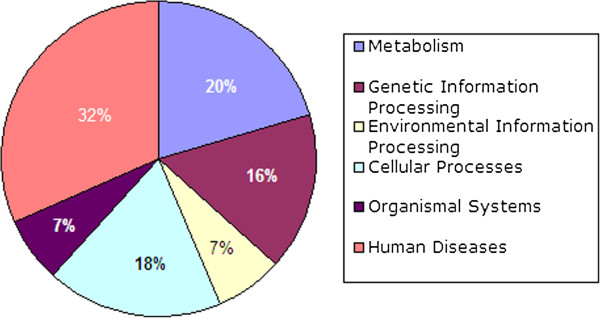
**KEGG pathway interactions for predicted proteases of the tapeworm,** ***Taenia solium.*** Graphic showing the relative proportions of proteases engaged in diverse signal processes and pathways. (Detailed information is provided in Additional file 2).

Most of the protein sequences, which have high sequence identity with those well-described protease in MEROPS database, were confirmed as having a conserved protease-specific domain (Additional file 1). Among them, we were able to assign orthology and KEGG (Kyoto Encyclopedia of Genes and Genomes) functional pathways to 117 *T. solium* proteases using KAAS analysis (Figure 1; the full annotation of KEGG pathways available in Additional file 2). Thirty-seven proteases were predicted engage in human pathogenesis, while 24 were predicted to be involved in metabolic pathways. Twenty-one proteases were predicted to be involved in cellular processes such as energy transport, cell cycle and communication, 19 proteases may play roles in genetic information processes and eight proteases likely perform functions in environmental information processes and organismal systems. Although almost all the proteases identified here have active sites, we caution that none have been shown experimentally to be catalytically active. We focused our discussion on several important regulatory proteases, observed for the first time in *T. solium* because this aspect offers obvious potential for targets of novel chemotherapies or the candidates for new vaccines [24, 25].

### Aspartic proteases

Aspartic proteases are important hydrolytic enzymes in medicinal chemistry because many of their members have become therapeutic targets for HIV/AIDS, Alzheimer’s disease, and other conditions. The catalytic activity of these proteins is driven by the Asp dyad, a pair of active site residues Asp residues participating in the hydrolysis of the substrate [26]. Twenty-four loci encoding aspartic proteases belonging to four families were identified in the tapeworm (Additional file 1). In family A2, 18 aspartic proteases were found. Among these 18, by using two prediction methods, TMMOD and TMHMM, we predicted that two proteases contained signal peptide sequences, and two proteases possessed a signal transmembrane (TM) domain. In family A1, we identified a single cathepsin D-like aspartic protease (LongOrf.asmbl_10039 Scaffold00045) that contains a signal peptide sequence. This hydrolase did not appear to include TM domains (Table 1; Additional file 1). All members of the family A2 have the highly conserved sequence and same active site. The active site of aspartic acid residues occur within a motif (Asp-Thr/Ser-Gly), in like fashion to pepsin [27, 28].

Secreted aspartic proteases (Saps) are common in eukaryotes. These kinds of enzymes represent the major virulence factors in human candidiasis and other fungal diseases, and participate in a wide range of fungal physiological processes as well as other fungal-host interactions. Saps are potential targets for the development of novel anti-fungal drugs [29]. In addition, aspartic proteases have attracted a great deal of interest as drug targets for malaria (*Plasmodium falciparum*) and related other protozoal diseases [30]. Both schistosomes and hookworms deploy cathepsin D within the gut of the adult worms to digest hemoglobin released from ingested host blood cells [31]. Although related information for functions of aspartic proteases cestodes is not yet available, the findings presented here provide insights on designs for novel drugs for cysticercosis and taeniasis.

### Cysteine proteases

Cysteine proteases play indispensable roles in cell biology of parasites [32, 33], but their functions in cestodes remain poorly characterized. Important parasite proteases are grouped among family C1 (cathepsin B and cathepsin L-like), family C2 (calpain-like) and other families [34]. Based on significant similarity to known cysteine proteases, 50 loci were detected in this study, of which 10% (5/50) had an identifiable signal sequence and thus are accessible to the secretory pathway. There was a slight discrepancy between the two TM domain prediction algorithms: TMMOD found a TM domain in three cysteine proteases, whereas TMHMM detected a TM domain in six members of this catalytic class (Table 1). In family C1, eight proteases were observed that contain cysteine-type cathepsin activity known to be involved in digestion of host proteins [35] (Additional file 1). Two of these loci encoded the cathepsin B domain. Cathepsin L-like cysteine proteases from the metacestode stage of *T. solium* induce serological responses during cysticercosis [15]; further investigation is recommended to establish their value of vaccine candidates [15]. Other studies have demonstrated that cathepsin B proteases play critical roles in the physiology of the carcinogenic liver fluke *Opisthorchis viverrini*[36], and related family enzymes can be targeted for development of therapeutic inhibitors or vaccination for control of fasciolosis [37].

**Figure 2:**
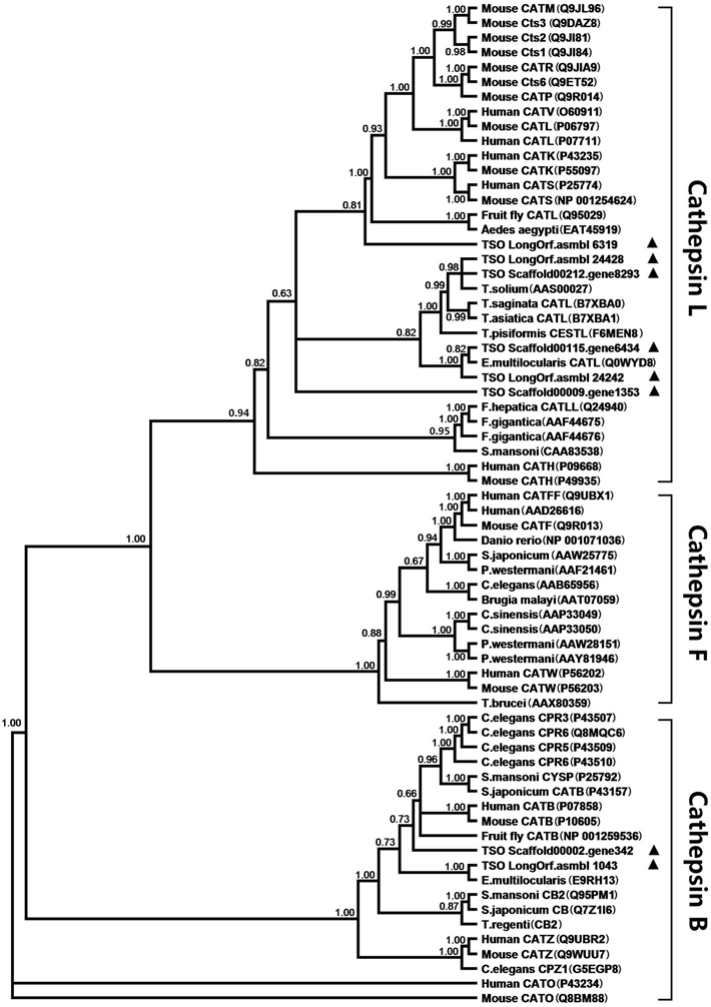
**Inferred phylogenetic relationships based on amino acid sequences of selected C1 family proteases.** The proteases of *Taenia solium* identified here are indicated with black triangle. Posterior support values are given at node (posterior probability >50%).

Phylogenetic relationships of C1 proteases (cathepsins) were analyzed using the orthologues from human, mouse, *Drosophila melanogaster*, *C. elegans*, *S. mansoni*, *S. japonicum Echinococcus multilocularis*, *T. solium* and three additional *Taenia* species - *T. saginata*, *T. asiatica*, and *T. pisiformis*. Phylogenetic trees revealed six proteases in *T. solium* that are cathepsin L or cathepsin L-like, and two proteases that are cathepsin B-like. However, cathepsin F proteases were not observed in putative proteome of *T. solium*. It is clear that two *T. solium* proteases (Scaffold00002.gene342, and LongOrf.asmbl 1043) are cathepsin B-like proteases, and one of them is closely related to the cathepsin B-like peptidase of *E. multilocularis* (EmCBP1: E9RH13). These cathepsin B-like proteases constitute a clade within the papain-like cysteine protease family, including homologues from schistosomes, *C. elegans* (CPR6, CPR3, CPR5 and CPZ1), human and mouse (CATB and CATZ), which is consistent with that of the CDD analysis. Six *T. solium* C1 proteases are cathepsin L or cathepsin L-like cysteine peptidases; among them, one protease (LongOrf.asmbl 6319) has a close relationship with a cathepsin L-like protease of the fruit fly (Q95029), and cathepsin L of human (such as CATS: P25774, CATK: P43235, CATL: P07711) and mouse (such as CATS: NP_001254624, CATK: P55097, CATL: P06797, CATM: Q9JL96, and so on). Four *T. solium* proteases clustered with CATL (cathepsin L-like cysteine peptidase) of *T. saginata*, *T. asiatica*, *T. pisiformis* and *E. multilocularis*, which deviates slightly from two *T. solium* proteases (Scaffold00009.gene1353 and LongOrf.asmbl 6319). These cathepsin L or cathepsin L-like proteases and cathepsin H of mouse and human branched together in a clade discrete from cathepsin F. Although the remaining three C1 cysteine proteases (LongOrf.asmbl_24428 Scaffold01127, Scaffold00212. gene8293, LongOrf.asmbl_24242 Scaffold00809) are not included in the phylogenetic analysis because their sequences were truncated apparently, it indicated a functional divergence among these *T. solium* C1 proteases (Figure 2).

In addition to digestive enzymes characterized as cathepsins, other abundant regulatory cysteine proteases identified in the genome of *T. solium* included calpain and caspase proteases. Calpain proteases are important calcium-dependent proteases that belong to the C2 family. Here we observed six members of the C2 family in the *T. solium* genome. Calpains perform a variety of functions in cytoskeletal remodeling processes, cell differentiation, apoptosis, and signal transduction [38]. Although reports on vaccine efficacy of calpain in tapeworm infections have yet to be published, calpains are under investigation as vaccine candidates against *S. japonicum* and *S. mansoni* where reductions in worm burden and egg production have been achieved by immunization [39, 40].

Caspases (interleukin-1 beta converting enzyme [ICE] homologues; cysteine-dependent aspartate-directed proteases) are well known for their roles in apoptosis (programmed cell death) in a wide range of organisms, including in platyhelminths [41–44]. In addition, inflammatory caspases mediate inflammation, immunity, and maturation and differentiation of certain cells including microglia and keratinocytes [45]. Despite a large overlap, caspases can be classified into three types: (1) initiator caspases participate in the upstream steps of the signaling cascade and can activate other signaling proteins, (2) effector caspases, which can lyse cellular proteins directly and this process ultimately results in classical signs of apoptosis, and (3) pro-inflammatory caspases, which activate the inflammatory cytokines [46]. Six *T. solium* caspases were identified here, and five have the conserved active site of Ala-Cys and/or His-Gly (Additional file 1). In comparison, *S. mansoni* has four caspases loci, of which exhibit conserved catalytic residues. *C. elegans* has four caspase loci, three of which have conserved catalytic residues. Induction of apoptosis in developing embryos is a potential approach for therapeutic intervention against nematodes [47]. Caspases are important regulatory proteins and are targets of chemotherapeutic agents against several diseases [48, 49].

We putatively identified 4 and 21 members of the C12 and C19 families of cysteine proteases, respectively. The C12 and C19 families also contain biochemically important enzymes containing ubiquitin hydrolase, which interacts with ubiquitin (Ub). Ubiquitin carboxyl-terminal hydrolase (UCH) proteins (e.g. UCH37) are involved in the deubiquitinating activity in the 19S or 26S proteasome regulatory complex. UCH enzymes play a crucial role in signaling pathways and in cell-cycle regulation [50]. These intracellular peptidases remove ubiquitin from polyubiquinated peptides by cleavage of isopeptide bonds. They hydrolyze bonds involving the carboxyl group of the C-terminal Gly residue of ubiquitin. De-ubiquitination edits the ubiquitin conjugates, which may ensure rescue from degradation, as well as recycling of the ubiquitin. The ubiquitin/proteasome system is responsible for most protein turnover in the mammalian cell [51, 52].

### Metalloproteases

Metalloproteases are highly conserved in evolution. They mediate the hydrolysis of bioactive peptides and extracellular matrix proteins. Metalloproteases require metal (usually zinc) ions for catalysis. A full-length cDNA termed TsSte24p has been reported, which appeared to encode a type I CaaX protease of the *T. solium* metacestode (TsM). The TsSte24p gene occurs as a single copy within the TsM genome, is constitutively expressed from metacestode to adult stages, and shares significant sequence identity with the type I CaaX protease of *Saccharomyces cerevisiae* Ste24p and *C. elegans* CeFACE-1 [53, 54]. Here, we determined that metalloproteases contribute a large proportion of proteolytic enzymes in the *T. solium* genome -- 69 loci were identified. 16% of these metalloproteases contained signal sequences and 27% exhibited one or more transmembrane alpha helices, indicating that these tapeworm metalloproteases are membrane bound (Table 1).

Our analysis indicated that 11 members of the M1 family are encoded by the tapeworm genome. Family M1 metalloproteases are dependent on a single zinc ion for activity, and all members of this family cleave the N-terminus residues of polypeptides. Indeed, many are aminopeptidases. The catalytic zinc ion is bound by two histidines and a glutamate. The histidines are situated within the HEXXH motif on one long helix with the glutamate on another antiparallel helix. The catalytic mechanism involves activation of a water molecule by the zinc ion. The glutamate of HEXXH is critical for catalysis and a tyrosine may also be involved [55]. The insect aminopeptidase A is the receptor for the insecticidal CrylAc toxin of *Bacillus thuringiensis*[56]. Similar studies have not yet been reported on these types of proteases in tapeworms, and their physiological roles in *T. solium* remain to be determined.

Numerous proteins operate in the mitochondria. The mitochondrial intermediate protease (MIP) and mitochondrial processing protease (MPP) often function in concert to cleave transit peptides from immature mitochondrial proteins synthesized in the cytoplasm [12, 57]. We identified a peptidase MIP belonging to the M3 superfamily. Like other metalloproteases in subclan MA (E), the members in the family M3 contain the HEXXH motif that forms the active site in conjunction with a carboxyl Glu residue. A single zinc ion is ligated by the sidechains of the two His residues, and the more COOH-terminal Glu. The members of the family M3 catalyze various peptidase reactions, including an unusual form of endopeptidase activity that is restricted to substrates of less 19 amino acid residues, with a particular preference for scission proximal to the C-terminus [58]. Another form of MIP peptidase that cleaves N-terminal octapeptides from proteins during import into the mitochondrion differs from bacterial peptidyl-dipeptidase Dcp and liberates C-terminal dipeptides [59].

ATP-dependent mitochondrial proteases are known to possess a wide variety of cellular associated activities. They play an essential role in quality control, turnover, and assembly of the respiratory chain complex proteins [60]. Three members of ATP-dependent proteases of the M41 family were identified in the genome of *T. solium*, and three contained an ATP binding motif with a conserved ATP binding site. Although related functions have not been described in *T. solium*, earlier reports demonstrated that metalloprotease inhibitors can cause paralysis of adult worms of *S. mansoni*[61], in similar fashion to humans and mice.

M50 family proteases contain metallo-endopeptidases, including the mammalian S2P [sterol regulatory element-binding protein (SREBP) Site-2 protease, S2P] proteases (subfamily M50B), and bacterial SpoIVFB (subfamily M50A). In this study, we observed one protease of the M50A subfamily possess the HEXXH catalytic motif (LongOrf.asmbl_14141 Scaffold00087). There are presumably six transmembrane helices (using TMHMM method) within this protease, in which the putative active site is located in the third transmembrane helix (around residues 175–195; Additional file 1). This result is consistent with previous reports that cleavages catalyzed by members of family M50 occur within or close to membranes [62]. S2P peptidase cleaves a Leu-Cys bond in the first transmembrane helix of the substrate through releasing the N-terminal transcription factor domain from membrane-bound SREBPs [63].

### Serine proteases

Forty-one serine proteases were predicted encoded within the genome of *T. solium*. They were classified into eight families. Thirteen loci were predicted to have one signal sequence, and five and four of them belong to the S1 and S8 family, respectively, in accord with the general understanding that the major members of S1 family proteases enter the secretory pathway via an N-terminal signal sequence. Nineteen of the serine proteases of *T. solium* possess at least one transmembrane alpha helix predicted by TMHMM (Table 1). The members in the S1 family of proteases possess a broad range of functions. Almost all S1 family members contain the catalytic triad His, Asp and Ser residues [64]. Although the catalytic serine residue is conserved for this protein among most vertebrates, previous studies have determined that the serine has been replaced by threonine in some not-peptidase paralogues, for example in human testes-specific protein TSP50 [65]. There are also many other non-peptidase homologues in which catalytic residues have been replaced [66].

**Figure 3:**
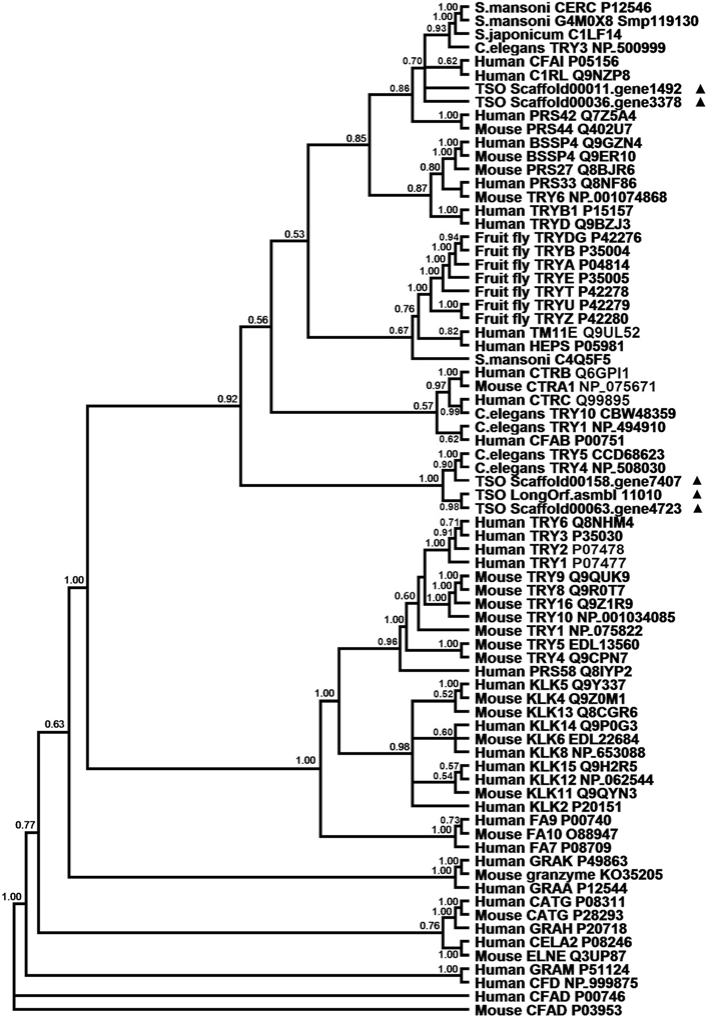
**Phylogenetic tree inferred from amino acid sequences of selected S1 family proteases.** The proteases of *Taenia solium* identified here are indicated with black triangle. Posterior support values are given at node (posterior probability >50%).

Within family S1, there are three main types of protease activity: 1) trypsin-like, where there is cleavage of amide substrates following Arg or Lys at P1 position; 2) chymotrypsin-like, where cleavage occurs following one of the hydrophobic amino acids at P1; and 3) elastase-like, with cleavage following an Ala at P1. These enzymes are usually synthesized as inactive precursor zymogens that are cleaved to generate their active forms in the case of activation sites being recognized during limited proteolysis. Nine S1 proteases were identified in *T. solium*; however, two of them did not exhibit significant similarity to the conserved protease domain (Additional file 1). Moreover, sequence alignment showed that four of these predicted proteins (LongOrf.asmbl_11010 Scaffold00053, Scaffold00011. gene1492 Scaffold00011, Scaffold00036.gene3378 Scaffold00036, Scaffold00158. gene7407 Scaffold00158) have the conserved catalytic triad of His, Asp, and Ser. It is notable that Ser was replaced by Thr in one hydrolase (Scaffold00063.gene4723 Scaffold00063), as occurs in the human TSP50 protease [65] (Additional files 1 and 3). These five proteases are trypsin-like serine proteases. Phylogenetic relationships of S1 proteases were analyzed using informative orthologues from human, mouse, *Drosophila*, *C. elegans*, *Schistosoma* and *T. solium*. One tapeworm protease (Scaffold00158. gene7407) clustered with TRY4 and TRY5 of *C. elegans*, and constituted an independent clade with other two proteases (LongOrf.asmbl 11010 and Scaffold00063. gene4723). Two other *T. solium* S1 proteases (Sscaffold00011. gene1492 and Sscaffold00036. gene3378) grouped adjacent to complement factor I light chain (P05156) and Complement C1r subcomponent-like protein (Q9NZP8) of human, TRY3 (NP_500999) of *C. elegans* and related schistosome enzymes. Although it is premature to define the functions of these five *T. solium* S1 proteases through the phylogenetic analysis, these results indicated that functional divergence might exist among the S1 proteases in *T. solium* (Figure 3).

Among the S1A subfamily, two proteases containing several LDLa (Low Density Lipoprotein Receptor Class A) domains were observed. LDLa is a cysteine-rich repeat domain that plays a central role in metabolism of mammalian cholesterol, especially during the receptor protein binds LDL [67]. It enters the cell by endocytosis [68]. Successive cysteine-rich repeats of ~ 40 residues are located at the amino-terminus of this multi-domain membrane protein. Here we observed that two putative proteases (Scaffold00025.gene2771 Scaffold00025 and Scaffold00005.gene846 Scaffold00005) contained 3 and 17 LDLa domains, respectively. These proteases might play a central role in cholesterol metabolism in this tapeworm. For the LDLa domain, the binding of calcium is required for in vitro formation of the native disulfide isomer and is necessary in establishment and maintenance of the modular structure [69].

In addition, two proteases from the S1B subfamily containing PDZ domains (Additional file 1) were present. One shares identity with cd00987 subfamily (CDD) and the other with cd00992 subfamily (CDD). PDZ domains occur in a variety of eumetazoan signaling molecules, often in tandem arrangements. The domains may be responsible for specific protein-protein interactions because most of them can bind to C-terminal polypeptides, internal (non-C-terminal) polypeptides and even lipids. In the cd00987 subfamily, protease-associated PDZ domains of C-terminal beta-strand form the peptide-binding groove base, a circular permutation with respect to PDZ domains was observed in signaling proteins, whereas in cd00992, the peptide-binding groove base is formed from N-terminal beta-strand [70, 71]. Six tapeworm members in the S41 family also contained the PDZ domain.

Members of peptidase family S8 include the serine endopeptidase subtilisin, which has a catalytic mechanism that is distinct from typical chymotrypsins. The S8 family has an Asp/His/Ser catalytic triad similar to that in trypsin-like proteases, but does not share the three-dimensional structure and is not homologous to trypsin. In the S8 family, serine acts as a nucleophile, aspartate as an electrophile, and histidine as a base, as members in the S1, S9 and S10 families [55]. The S8 family includes two subfamilies, subtilisin and kexin being type-examples for subfamily S8A and S8B, respectively. Tripeptidyl-peptidase II (TPP-II) is a divergent example of S8A subfamily. We identified 13 members of the S8 family, of which two belong to S8A, four belong to S8B and seven members belong to others (Additional file 1). In the S8A subfamily, one member is a SKI-1-like (type I membrane-bound subtilisin-kexin-isoenzyme) protein, which is a secretory Ca^2+^-dependent serine protease that cleaves at nonbasic residues: Thr, Leu and Lys. SKI-1 plays a critical role in the regulation of the synthesis and metabolism of cholesterol and fatty acids [72]. The S8A enzyme tripeptidyl aminopeptidases_II cleaves tripeptides from the free N terminus of oligopeptides; it also exhibits endoproteolytic activity [73]. In the S8B subfamily, all four members are kexin_furin-like convertases contain an Asp/His/Ser catalytic triad that is discrete from that of trypsin. Kexins participate in the activation of peptide hormones, growth factors, and viral proteins [74]. Furins are involved in the tissue remodeling of cardiovascular in the *trans*-Golgi Network (TGN), in endosomes or at cell surface through cleavage of cell surface vasoactive peptides and proteins. Furins also play a key role in blood pressure regulation by the activation of transforming growth factor (TGF)-beta [75, 76]. The functions of kexins and furins of *T. solium* remain to be determined.

It is noteworthy that seven members (Scaffold00006.gene1002 Scaffold00006, Scaffold00007.gene1037 Scaffold00007, Scaffold00007.gene1092 Scaffold00007, Scaffold00008.gene1266 Scaffold00008, Scaffold00009.gene1307 Scaffold00009, Scaffold00038. gene3515 Scaffold00038, Scaffold00003.gene565 Scaffold00003) of the S8 family contain a large number of (4–26) of cadherin tandem repeat domains. Cadherins are glycoproteins involved in Ca^2+^-mediated cell-cell adhesion [77]. The cadherin repeat domains often exist as tandem repeats in the extracellular regions; they may mediate cell-cell contact when bound to calcium. They play numerous roles in cell fate, signalling, proliferation, differentiation, and migration. Cadherin-repeat containing proteins exist as monomers, homodimers, or heterodimers [55, 78, 79]. Interestingly, one of these tapeworm proteases not only contained 26 cadherin_repeat domains, but also possessed two calcium-binding EGF-like domains. EGF_CA domains, present in a large number of membrane-bound and extracellular proteins, play a crucial role in numerous protein-protein interactions [80]. Although exact functions of these cestode enzymes are not clear, potential roles during the parasite-host interaction, such as parasite invasion, adherence, survival and growth, can be predicted.

Family S54 – the rhomboid proteases – includes membrane-bound serine endopeptidases. The hydrolases separate bioactive signaling peptides from anchoring TM domains. The rhomboid proteases are widely distributed among bacteria, archaea and eukaryotes [81]. Rhomboid proteases are critical during embryogenesis in *D. melanogaster*, and parasite-encoded rhomboid enzymes play important roles in invasion of host cells by *Toxoplasma gondii* and malaria parasites [82]. In the *T. solium* genome, one member belonging to the S54 family has six TM helices predicted by both TMMOD and TMHMM showing a likely conserved structure among taxa (Additional file 1). However, we were not able to locate the deduced active site of the protease (which may reflect inaccurate sequencing). It is noteworthy that this protease contains an EF-hand, calcium-binding motif with calcium sensors and calcium signal modulators. Ca^2+^ binding induces a conformational change in the EF-hand motif, leading to the activation or inactivation of target proteins [83].

We characterized a single AAA mitochondrial protease of the S16 family. These kinds of proteases are known to exhibit numerous regulatory activities, including selective degradation of misfolded, unassembled or oxidatively damaged polypeptides in the mitochondrial matrix, chaperone functions in the assembly of inner membrane protein complexes, regulation functions on mitochondrial gene expression and safeguard functions for the integrity of the mitochondrial genome, through binding to mitochondrial promoters and RNA. Down-regulation of this protease causes a general activation of caspases and leads to apoptosis [84, 85]. *T. solium* also has two S26 family members, which may be responsible for processing precursor proteins to mature forms [86].

### Threonine proteases

Threonine proteases are closely associated with the elements of the 20S proteasome [12]. The proteasome complex is comprised of four rings of seven subunits, which form a hollow cylinder, with the active sites located on the inner walls of the chamber [87]. Rings one and four contain alpha-type subunits whereas rings two and three are composed of beta-type subunits. The N-terminal threonine residues of some beta subunits are the nucleophiles in catalysis. In the eukaryotic proteasome, only the three kinds of beta subunits in ring three possess catalytic activity [88]. The majority of threonine proteases identified here in *T. solium* appear to be subunits of the proteasome, and seven alpha subunits and seven beta subunits of the proteasome were observed. We also identified a taspase-like protease, an endopeptidase that cleaves specific substrates following aspartate residues, and a glycosylasparaginase in the *T. solium* genome. Mature forms of taspases exhibit endopeptidase activity, and regulate transcription of many genes through hydrolysis of the TFIIA transcription factor [89].

## Conclusions

Bioinformatic techniques were used to explore the putative proteins encoded by the newly reported genome of *T. solium* for sequences homologous to proteases. Through comprehensive analysis, 200 predicted proteases were identified and >98% of them are reported for the first time from *T. solium*. Aside from the three proteases described previously, altogether we determined 197 previously unidentified proteases, which likely participate in broad range of biological processes. Here we focused on regulatory proteases since they generally possess essential functions in the virulence - including invasion/entry, tissue migration and the suppression of host immune responses - and the developmental progression of the life cycle of this parasite. Whereas the significance of protease-mediated regulatory function needs to be established through experimentation, the annotation of the protease-encoding sequences of this tapeworm, particularly regulatory proteases, can be expected to provide leads and other information on chemotherapeutic targets and candidates for novel interventions against cysticercosis.

## Methods and data

Putative homologues of known proteases in the *T. solium* genome were identified using the complete set of core protease sequences from the MEROPS (release 9.7) database [55, 90]. They consist of a non-redundant library of the catalytic unit of a protease and exclude all other functional units, such as domains of Ca^2+^-binding and ATP-binding. These core sequences were used to avoid false positive identification of proteases due to high sequence identity in its non-catalytic parts. Core sequences were compared to predicted proteins from the annotated *T. solium* genome sequenced in our laboratories and in the Beijing Institute of Genomics, Chinese Academy of Sciences. We downloaded the complete database of predicted proteins of *T. solium* genome updated on November 2, 2012.

The MEROPS batch BLAST [91] comparisons were carried out using the putative proteins as the queries, and the MEROPS peptidases as the database, where predicted proteins were queried against all members of the protease database, and sequences with similarity scores (E-value) greater than 1e-04 were retained as *T. solium* protease homologs. For the initial batch BLAST results, query sequences, which are analogous to non-protease sequences (protease-like sequences but without active sites) were culled. In addition, predicted proteins that were shorter than 80 residues were removed. Comprehensive analyses were implemented on the remaining sequences as follows.

In order to characterize the sequences, analyses were conducted on the results from the MEROPS Batch BLAST query. Firstly, we examined the predicted function of *T. solium* sequences through searching for conserved motif and domains in the protein sequences independently. This was done using the Batch Web CD-search tool in the Conserved Domain Database (CDD) (version 2.25) of NCBI [92–95]. CDD searches employ a reverse position-specific BLAST (RPS-BLAST) to align query sequence to protein domains from SMART v. 7.0 [96], Pfam v. 26.0 [97, 98], and COG [99]. Secondly, pathway-based functional orthology of the dataset was classified using the KEGG (Kyoto Encyclopedia of Genes and Genomes) Automated Annotation Server (KAAS) [100]. Thirdly, alpha-helix domains that likely anchor a cellular membrane were predicted using two methods TMHMM (http://www.cbs.dtu.dk/services/TMHMM/) [101] and TMMOD [102]. Fourthly, because of the expected cellular location and potential to enter the secretory pathway of a cell are also helpful in classifying proteins, we identified the signal sequences in the predicted proteins with signalP 4.1 [103]. The D score is the most reliable score to discriminate valid signal sequences in proteins, which is a weighted average of the maximal Y scores (a combined cleavage site score to determine the most likely location of the cleavage site of the signal sequence) and the mean S-score (from position 1 to the position immediately before the maximal Y-score) [104]. In this study, proteins with D score greater than 0.50 were recognized as having an N-terminal signal sequence.

Sequence alignments were accomplished using Clustal X 1.81 [105]. The resulting alignments were subjected to phylogenetic analysis using MrBayes 3.1.2 under the default setting [106]. Two simultaneous were carried out, each being independent runs on each data set. In every case two runs, each of four chains, including three heated chains and one cold chain, was specified. MrBayes determined the most appropriate model (“prset aamodelpr = mixed”), and at least 10,000,000 generations were run and trees sampled every 1,000. Runs were continued until the average standard deviation of the split frequencies between the two runs was < 0.01. The first 25% of trees were omitted as burn-in prior to summarizing sampled trees. Summarizing samples produced a consensus tree with branch bifurcation support (clade credibility) indicated. Clade credibility was calculated for each bifurcation as the proportion of sampled trees with that bifurcation [106, 107].

### Availability of supporting data

All phylogenetic data (alignments, phylogenetic trees, and relevant primary data) have been submitted in TreeBase with study ID 15682 (Study Accession URL: http://purl.org/phylo/treebase/phylows/study/TB2:S15682).

## Electronic supplementary material

Additional file 1: **Sequences of** ***Taenia solium*** **proteases sequences that have significant similarity and active site to known proteases.** The tables list *T. solium* sequences with share significant similarity to known proteases, protease family names, conserved domains, active sites, signal sequences and transmembrane regions. (DOC 710 KB)

Additional file 2: **KAAS analysis: KEGG pathway assignment and KEGG orthology number (KO number) for** ***Taenia solium*** **proteases.** Bioinformatic analysis using the Kyoto Encyclopedia of Genes and Genomes used to predict probable functions and the cellular processes *for* the tapeworm proteases, based on orthologous relationships of proteases for which functions in other species have been clearly established. (DOC 58 KB)

Additional file 3: **C1_S1 family catalytic residues - active sites shown in black of blue.** Partial sequence alignment of a family of proteases for several species; active site residues central to catalysis are highlighted. (DOC 78 KB)
