# Supplementary material for: Genome-wide analysis of regulatory proteases sequences identified through bioinformatics data mining in Taenia solium
Source: BMC Genomics. 2014 Jun 4;15:428. doi: 10.1186/1471-2164-15-428 (PMC4070553; doi:10.1186/1471-2164-15-428)
Supplement: Supplementary file 1 — Additional file 1: Sequences of Taenia solium proteases sequences that have significant similarity and active site to known proteases. The tables list T. solium sequences with share significant similarity to known proteases, protease family names, conserved domains, active sites, signal sequences and transmembrane regions. (DOC 710 KB) [file 12864_2013_6143_MOESM1_ESM.doc]

| **Family** | **Gene ID** | **Aspartic proteases Conserved domains** | **Domain family** | **E-Value** | **Active sites** | **Signal**  **sequence** | **TMHMN**  **helices** |
| --- | --- | --- | --- | --- | --- | --- | --- |
| A1A | LongOrf.asmbl_10039 Scaffold00045 | Cathepsin D-like | [cd05485](http://www.ncbi.nlm.nih.gov/entrez/query.fcgi?cmd=Search&doptcmdl=GenPept&db=cdd&term=cd05485) | 2.95E-168 | D88, Y133, D274 | 16 |  |
| A2A | Scaffold00013.gene1644 Scaffold00013 | Retropepsins; pepsin-like | cd00303 | 3.14E-11 | D306 |  |  |
|  | Scaffold00024.gene2637 Scaffold00024 | Retropepsins; pepsin-like | cd00303 | 1.44E-08 |  | 45 |  |
|  | Scaffold00053.gene4324 Scaffold00053 | Retropepsins; pepsin-like | cd00303 | 6.61E-10 | D257 |  |  |
|  | Scaffold00010.gene1446 Scaffold00010 | Retropepsins; pepsin-like | cd00303 | 2.05E-09 | D191 |  |  |
|  | Scaffold00013.gene1691 Scaffold00013 | Retropepsins; pepsin-like | cd00303 | 1.19E-08 | D63 |  |  |
|  | Scaffold00116.gene6445 Scaffold00116 | Retropepsins; pepsin-like | cd00303 | 4.30E-09 | D213 |  |  |
|  | Scaffold00136.gene6963 Scaffold00136 | Retropepsins; pepsin-like | cd00303 | 5.85E-05 | D213 |  |  |
|  | Scaffold00155.gene7338 Scaffold00155 | Retropepsins; pepsin-like | cd00303 | 1.42E-08 | D298 |  |  |
|  | Scaffold00222.gene8405 Scaffold00222 | Retropepsins; pepsin-like | cd00303 | 2.19E-10 | D174 | 25 | 1 |
|  | Scaffold00293.gene9160 Scaffold00293 | Retropepsins; pepsin-like | cd00303 | 3.54E-09 | D89 |  |  |
|  | Scaffold00443.gene9836 Scaffold00443 | Retropepsins; pepsin-like | cd00303 | 4.51E-08 | D84 |  |  |
|  | Scaffold00607.gene10168 Scaffold00607 | Retropepsins; pepsin-like | cd00303 | 1.01E-09 | D252 |  |  |
|  | Scaffold00609.gene10171 Scaffold00609 | Retropepsins; pepsin-like | cd00303 | 2.59E-07 | D182 |  |  |
|  | Scaffold01070.gene10611 Scaffold01070 | Retropepsins; pepsin-like | cd00303 | 1.90E-09 | D338 |  | 1 |
|  | Scaffold01299.gene10720 Scaffold01299 | Retropepsins; pepsin-like | cd00303 | 2.04E-06 | D84 |  |  |
|  | Scaffold01695.gene10847 Scaffold01695 | Retropepsins; pepsin-like | cd00303 | 3.60E-07 | D58 |  |  |
|  | Scaffold03691.gene11201 Scaffold03691 | Retropepsins; pepsin-like | cd00303 | 6.75E-09 | D84 |  |  |
|  | Scaffold00287.gene9105 Scaffold00287 | Retropepsins; pepsin-like | cd00303 | 6.88E-11 | D213 |  |  |
|  |  | RT_LTR | cd01647 | 6.85E-32 | D482-Y487 |  |  |
|  |  | RVT_1 | pfam00078 | 7.28E-08 |  |  |  |
| A11 | Scaffold00001.gene83 Scaffold00001 | RNase_H superfamily | cd09274 | 4.54E-19 | D19, G62D |  |  |
|  | Scaffold00071.gene5019 Scaffold00071 | RNase_H superfamily | cd09274 | 1.29E-08 | Y24, R38 |  |  |
| A22 | LongOrf.asmbl_10976 Scaffold00053 | Signal peptidase | pfam04258 | 8.90E-58 |  |  | 8 |
|  | LongOrf.asmbl_826 Scaffold00002 | Presenilin | [pfam01080](http://www.ncbi.nlm.nih.gov/entrez/query.fcgi?cmd=Search&doptcmdl=GenPept&db=cdd&term=pfam01080) | 9.93E-108 |  |  | 7 |
|  | Scaffold00016.gene1964 Scaffold00016 | Signal peptidase | pfam04258 | 2.00E-29 |  | 36 | 10 |

Additional file 1

S1: aspartic proteases

S1: cysteine proteases

| **Family** | **Gene ID** | **Cysteine proteases**  **Conserved domains** | **Domain family** | **E-Value** | **Active sites** | **Signal**  **sequence** | **TMHMN**  **helices** |
| --- | --- | --- | --- | --- | --- | --- | --- |
| C01A | LongOrf.asmbl_1043 Scaffold00002 | Peptidase_C1A_CathepsinB | cd02620 | 3.04989e-122 | Q124, C130, H303, N323 | 23 |  |
|  | Scaffold00002.gene342 Scaffold00002 | Peptidase_C1A_CathepsinB | cd02620 | 5.65362e-110 | Q164, C170, H340, N360 |  |  |
|  | LongOrf.asmbl_24428 Scaffold01127 | Peptidase_C1A, papain | cd02248 | 1.88192e-37 | Q142, C148 | 17 |  |
|  | LongOrf.asmbl_6319 Scaffold00021 | Peptidase_C1A, papain | cd02248 | 1.97601e-97 | Q146, C152, H297, N317 | 17 |  |
|  | Scaffold00212.gene8293 Scaffold00212 | Peptidase_C1A, papain | cd02248 | 2.5201e-45 | H94, N114 |  |  |
|  | Scaffold00115.gene6434 Scaffold00115 | Peptidase_C1A, papain | cd02248 | 6.1174e-101 | Q139, C145, H283, N304 | 20 | 1 |
|  | LongOrf.asmbl_24242 Scaffold00809 | Peptidase_C1A, papain | cd02248 | 7.47932e-85 | H138, N159 |  |  |
|  | Scaffold00009.gene1353 Scaffold00009 | Peptidase_C1A, papain | cd02248 | 2.27735e-62 | Q165, C171, H307, N328 |  | 1 |
| C02A | LongOrf.asmbl_4585 Scaffold00013 | Calpains, domains II | cd00044 | 1.46991e-97 | Q144, C150, H308, N332 |  |  |
|  |  | Calpain_III | cd00214 | 2.31804e-47 | T444E, D445E, P446E, C455 |  |  |
| C02A | LongOrf.asmbl_13767 Scaffold00083 | Calpains, domains II | cd00044 | 5.53272e-72 | Q269, C275, H444, N464 |  |  |
| C02A | LongOrf.asmbl_13200 Scaffold00076 | Calpains, domains II | cd00044 | 2.67266e-76 | H160, N189 |  |  |
| C02A | Scaffold00130.gene6821 Scaffold00130 | Calpains, domains II | cd00044 | 6.45281e-53 | Q158H, X186N |  |  |
| C02A | Scaffold00049.gene4123 Scaffold00049 | Calpains, domains II | cd00044 | 1.74275e-84 | C27, H184, N208 |  |  |
| C02A | Scaffold00078.gene5230 Scaffold00078 | CysPc superfamily | [smart00230](http://www.ncbi.nlm.nih.gov/entrez/query.fcgi?cmd=Search&doptcmdl=GenPept&db=cdd&term=smart00230) | 4.14958e-28 | Y135H, S175N |  |  |
| C12 | LongOrf.asmbl_4936 Scaffold00014 | Peptidase_C12_UCH_L1_L3 | cd09616 | 1.6818e-76 | Q78, C84, H158, D173 |  |  |
| C12 | LongOrf.asmbl_7418 Scaffold00026 | Peptidase_C12_UCH37_BAP1 | cd09617 | 5.63514e-86 | Q79, C95, H161, D176 |  |  |
| C12 | Scaffold00017.gene2074 Scaffold00017 | Peptidase_C12 superfamily | cd09617 | 4.34699e-26 | Q184, C190, H332, D347 |  | 0 |
| C14A | LongOrf.asmbl_3186 Scaffold00007 | Caspase | cd00032 | 6.56043e-70 | H235, C278 |  |  |
| C14A | Scaffold00004.gene651 Scaffold00004 | Caspase | cd00032 | 1.46102e-80 | H176, C218 |  |  |
| C14A | Scaffold00079.gene5290 Scaffold00079 | Caspase | cd00032 | 2.79893e-63 | H554, C597 |  |  |
| C14A | LongOrf.asmbl_16154 Scaffold00112 | Caspase | [cd00032](http://www.ncbi.nlm.nih.gov/entrez/query.fcgi?cmd=Search&doptcmdl=GenPept&db=cdd&term=cd00032) | 2.43612e-39 | H172 |  |  |
| C14A | Scaffold01059.gene10604 Scaffold01059 | Caspase | [cd00032](http://www.ncbi.nlm.nih.gov/entrez/query.fcgi?cmd=Search&doptcmdl=GenPept&db=cdd&term=cd00032) | 1.52865e-23 |  |  | 1 |
| C14A | Scaffold01059.gene10605 Scaffold01059 | Caspase | [smart00115](http://www.ncbi.nlm.nih.gov/entrez/query.fcgi?cmd=Search&doptcmdl=GenPept&db=cdd&term=smart00115) | 4.94532e-09 | C36 |  | 1 |
| C15 | Scaffold00125.gene6704 Scaffold00125 | Peptidase_C15 superfamily | [cd00501](http://www.ncbi.nlm.nih.gov/entrez/query.fcgi?cmd=Search&doptcmdl=GenPept&db=cdd&term=cd00501) | 1.17281e-26 | E109, C166, S189H |  |  |
| C19 | LongOrf.asmbl_21266 Scaffold00227 | Peptidase_C19 | cd02257 | 1.92485e-15 | N73, C78, H470, D495 |  | 1 |
|  | LongOrf.asmbl_20169 Scaffold00194 | Peptidase_C19 | cd02257 | 1.6277e-21 | H429, D446 |  | 1 |
|  | LongOrf.asmbl_8072 Scaffold00031 | Peptidase_C19 | cd02257 | 1.92824e-07 | N45, C50, H726, D744 |  |  |
|  | Scaffold00054.gene4346 Scaffold00054 | Peptidase_C19 | cd02257 | 2.19832e-05 | H442, D459 |  |  |
|  | Scaffold00156.gene7356 Scaffold00156 | Peptidase_C19 | cd02257 | 1.41141e-05 | D513 |  |  |
|  |  | Peptidase C19 | [cd02659](http://www.ncbi.nlm.nih.gov/entrez/query.fcgi?cmd=Search&doptcmdl=GenPept&db=cdd&term=cd02659) | 3.12e-29 | H94, D112, |  |  |
|  | LongOrf.asmbl_23107 Scaffold00348 | Peptidase_C19E | cd02661 | 5.35475e-74 | N158, C163, H436, D452 |  |  |
|  | LongOrf.asmbl_1417 Scaffold00003 | Peptidase_C19D | [cd02660](http://www.ncbi.nlm.nih.gov/entrez/query.fcgi?cmd=Search&doptcmdl=GenPept&db=cdd&term=cd02660) | 3.62989e-38 | H701, D718 |  |  |
|  |  | Peptidase_C19R | [cd02674](http://www.ncbi.nlm.nih.gov/entrez/query.fcgi?cmd=Search&doptcmdl=GenPept&db=cdd&term=cd02660) | 9.68e-26 | N247, C252 |  |  |
|  | LongOrf.asmbl_23799 Scaffold00500 | Peptidase_C19 | [cd02657](http://www.ncbi.nlm.nih.gov/entrez/query.fcgi?cmd=Search&doptcmdl=GenPept&db=cdd&term=cd02657) | 6.66489e-66 | N114, C119, H454, D470 |  |  |
|  | LongOrf.asmbl_23911 Scaffold00543 | Peptidase_C19R | [cd02674](http://www.ncbi.nlm.nih.gov/entrez/query.fcgi?cmd=Search&doptcmdl=GenPept&db=cdd&term=cd02674) | 1.99e-12 | N113, C118 |  |  |
|  |  | Peptidase_C19E | [cd02661](http://www.ncbi.nlm.nih.gov/entrez/query.fcgi?cmd=Search&doptcmdl=GenPept&db=cdd&term=cd02661) | 1.2238e-30 | H552, D575 |  |  |
|  | LongOrf.asmbl_24548 Scaffold01536 | Peptidase_C19D | [cd02660](http://www.ncbi.nlm.nih.gov/entrez/query.fcgi?cmd=Search&doptcmdl=GenPept&db=cdd&term=cd02660) | 4.1706e-47 | N168, C173, H532, D549 |  |  |
|  | Scaffold00061.gene4636 Scaffold00061 | Peptidase_C19C | [cd02659](http://www.ncbi.nlm.nih.gov/entrez/query.fcgi?cmd=Search&doptcmdl=GenPept&db=cdd&term=cd02659) | 2.01507e-69 | N2390, C2395, H2725, D2799 |  |  |
|  | Scaffold00051.gene4220 Scaffold00051 | Peptidase_C19B | cd02658 | 2.74422e-59 | N383, C388, H873, D914 |  |  |
|  | Scaffold00037.gene3444 Scaffold00037 | peptidase_C19C | cd02659 | 1.47752e-127 | N223, C228, H474, D492 |  |  |
|  | Scaffold00034.gene3256 Scaffold00034 | peptidase_C19C | cd02659 | 1.02669e-95 | N1797, C1802, H2104, D2126 |  |  |
|  | LongOrf.asmbl_3312 Scaffold00008 | peptidase_C19C | cd02659 | 1.71984e-132 | N343, C348, H589, D607 |  |  |
|  | LongOrf.asmbl_11082 Scaffold00054 | Peptidase_C19G | cd02663 | 7.28377e-144 | N28, C33, H304, D320 |  |  |
|  | LongOrf.asmbl_8663 Scaffold00036 | Peptidase_C19K | cd02667 | 3.47104e-37 | N22, C27, H636, D707 |  |  |
|  | LongOrf.asmbl_4731 Scaffold00013 | Peptidase_C19L | cd02668 | 2.08926e-94 | N88, C93, H340, D360 |  |  |
|  | LongOrf.asmbl_3982 Scaffold00010 | Peptidase_C19M | cd02669 | 0 | N188, D193, T478, D496 |  |  |
|  | Scaffold00186.gene7880 Scaffold00186 | Peptidase_C19R | cd02674 | 1.94693e-75 | N375, C380, H665, D683 |  |  |
|  | LongOrf.asmbl_11541 Scaffold00059 | Peptidase_C19R | cd02674 | 2.5601e-41 | H455, D473 |  |  |
| C44 | Scaffold00051.gene4200 Scaffold00051 | Glutamine amidotransferases | cd00714 | 1.94733e-76 | C2, R33, R119, W120, T122, H123, H132, N147, G148, D172, T173, |  | 0 |
|  |  | SIS_GlmS_GlmD_1 | cd05008 | 2.76139e-51 |  |  |  |
|  |  | SIS_GlmS_GlmD_2 | cd05009 | 2.14616e-24 |  |  |  |
| C44 | Scaffold00099.gene5950 Scaffold00099 | Glutamine amidotransferases | cd00713 | 5.30548e-172 | C44, R74, R253, N274, G275, E276, D316 | 15 |  |
|  |  | GltS_FMN  [Glutamate synthase (GltS) FMN-binding domain] | cd02808 | 3.80021e-132 | G1078, A1079G, M1080, S1081, E1008, Q1027, K1049, K1055, R1075, K1117, G1147, T1148, G1149, D1188, Q1190G, S1211G, T1212S, C1220, C1226, C1231 |  |  |
|  |  | GltB_C | cd00982 | 3.25757e-89 |  |  |  |
| C46 | Scaffold00067.gene4884 Scaffold00067 | Hint | cd00081 | 1.1993e-14 | C224, T298, H301 |  |  |
| C56 | LongOrf.asmbl_7288 Scaffold00026 | GATase1_DJ-1 | cd03135 | 1.71221e-39 | C104 |  |  |
| C56 | LongOrf.asmbl_13755 Scaffold00083 | GATase1_ES1 | cd03133 | 1.63883e-105 | C164 |  |  |

S3: Metalloproteass

| **Family** | **Gene ID** | **Metalloprotease**  **Conserved domains** | **Domain family** | **E-Value** | **Active sites** | **Metal binding site** | **Signal**  **sequence** | **TMHMN**  **helices** |
| --- | --- | --- | --- | --- | --- | --- | --- | --- |
| M01 | LongOrf.asmbl_23061 Scaffold00346 | M1_APN_2 | cd09601 | 0 | - | H353, H357, E376 |  |  |
|  |  | Peptidase_M1 | pfam01433 | 8.02E-158 |  |  |  |  |
|  | Scaffold00065.gene4802 Scaffold00065 | M1_APN_2 | cd09601 | 9.38E-176 | E230, Y315 | H229, H233, E252 |  |  |
|  | Scaffold00104.gene6118 Scaffold00104 | M1_APN_2 | cd09601 | 0 | - | H393, H397, E416 |  | 1 |
|  | Scaffold00263.gene8897 Scaffold00263 | M1_APN_2 | cd09601 | 0 | E325, Y410 | H324, H328, E347 |  |  |
|  | Scaffold00263.gene8901 Scaffold00263 | M1_APN_2 | cd09601 | 1.06E-179 | E263, Y348 | H262, H266, E285 |  |  |
|  | Scaffold00346.gene9478 Scaffold00346 | M1_APN_2 | cd09601 | 5.56E-92 | E129 | H128, H132, E151 |  |  |
|  | Scaffold00568.gene10114 Scaffold00568 | M1_APN_2 | cd09601 | 4.37E-146 | E179, Y264 | H178, H182, E201 |  |  |
|  | Scaffold00806.gene10404 Scaffold00806 | M1_APN_2 | cd09601 | 2.05E-141 | E127, Y212 | H126, H130, E149 |  |  |
|  | Scaffold00045.gene3907 Scaffold00045 | M1_LTA4H | cd09599 | 0 | Q148, Q150, A151, V307, YGGME283-287, H310, E311, H314, F329L, E333, E340, F393, Y398 | H310, H314, E333 |  |  |
| M03 | Scaffold00004.gene624 Scaffold00004 | Thermolysin-like proteinases | pfam01432 | 3.58E-22 | S562H, A563E, R566H, R718Y, E721Y | S552H, R556H |  | 4 |
|  |  | PAP2_containing_2_like | cd03391 | 9.18E-24 | K944, R951, S973, G974, H975, R1011, H1017, D1021 | - |  |  |
| M03A | LongOrf.asmbl_7835 Scaffold00029 | M3A_MIP(Mitochondrial intermediate peptidase) | [cd06457](http://www.ncbi.nlm.nih.gov/entrez/query.fcgi?cmd=Search&doptcmdl=GenPept&db=cdd&term=cd06457) | 0 | H515, H519, E516, E544, H669, Y677, Y680 | H515, H519, E544, |  |  |
| M10A | Scaffold00054.gene4368 Scaffold00054 | Matrix metalloproteinase | cd04278 | 2.17E-58 | E272, H271, H275, H281 | GVLAHAF226-232, D245, E272, H271, H281, P291, Y292, Y293 |  | 1 |
|  |  | Hemopexin-like repeats | cd00094 | 1.37E-34 | - | D357, I359, D414, A416, D465, V467, T520D, A522 |  |  |
| M12A | LongOrf.asmbl_12456 Scaffold00067 | ZnMc_astacin_like | cd04280 | 2.40E-46 | E125, H124, H128, H134, Y183 |  | 18, 19 |  |
| M12A | Scaffold00082.gene5370 Scaffold00082 | ZnMc_BMP1_TLD | [cd04281](http://www.ncbi.nlm.nih.gov/entrez/query.fcgi?cmd=Search&doptcmdl=GenPept&db=cdd&term=cd04281) | 2.01E-101 | E319, H318, H322, H328 | - |  |  |
|  |  | CUB | cd00041 | 1.04E-34 | T623S, A650R, S655Y, P692V, T716L, E718Y, Q720Y | polypeptide binding site |  |  |
|  |  | CUB | cd00041 | 1.37E-34 |  |  |  |  |
|  |  | CUB | cd00041 | 5.68E-33 |  |  |  |  |
|  |  | CUB | cd00041 | 2.83E-25 |  |  |  |  |
|  |  | CUB | cd00041 | 1.93E-06 |  |  |  |  |
|  |  | EGF_CA | cd00054 | 1.39E-06 | - | D878, E881Q, N895 |  |  |
| M12B | LongOrf.asmbl_2871 Scaffold00006 | TACE-like metalloprotease | [cd04270](http://www.ncbi.nlm.nih.gov/entrez/query.fcgi?cmd=Search&doptcmdl=GenPept&db=cdd&term=cd04270) | 1.22E-54 | E185, H184, H188, H194 | - |  | 1 |
| M12B | Scaffold00006.gene972 Scaffold00006 | TACE-like metalloprotease | [cd04270](http://www.ncbi.nlm.nih.gov/entrez/query.fcgi?cmd=Search&doptcmdl=GenPept&db=cdd&term=cd04270) | 2.35E-27 | E112, H111, H115, H121 |  |  | 1 |
| M12B | Scaffold00064.gene4768 Scaffold00064 | TACE-like metalloprotease | [cd04270](http://www.ncbi.nlm.nih.gov/entrez/query.fcgi?cmd=Search&doptcmdl=GenPept&db=cdd&term=cd04270) | 2.99E-59 | E536, H535, H539, H545 | - | 21, 22 | 1 |
| M12B | Scaffold00011.gene1489 Scaffold00011 | ADAMTS-like metalloprotease | [cd04273](http://www.ncbi.nlm.nih.gov/entrez/query.fcgi?cmd=Search&doptcmdl=GenPept&db=cdd&term=cd04273) | 1.69E-32 | E289, H288, H292, H298 | - | 22, 23 |  |
| M12B | Scaffold00003.gene496 Scaffold00003 | Adamalysin_II-like metalloprotease | cd04269 | 1.68E-17 | H45, E46, H49, H55 | - |  | 1 |
|  | Scaffold00168.gene7594 Scaffold00168 | Adamalysin_II-like metalloprotease | [cd04269](http://www.ncbi.nlm.nih.gov/entrez/query.fcgi?cmd=Search&doptcmdl=GenPept&db=cdd&term=cd04269) | 4.37E-13 | K435H, S436E, E439H | - | 24, 25 | 2 |
| M13 | Scaffold00122.gene6610 Scaffold00122 | M13 | cd08662 | 0 | N683, A684, V721, H724, E725, H728, E784, F828, A829, H850, R856 | H724, H728, E784 |  | 1 |
| M14A | LongOrf.asmbl_22677 Scaffold00308 | M14_CP_A-B_like | cd03860 | 4.16E-119 | H183, E186, R236, N253, R254, H306, Y359, A361, E382 | H183, E186, H306 | 19, 20 | 1 |
| M14B | LongOrf.asmbl_9553 Scaffold00042 | M14_CP_N-E_like | cd03858 | 1.59E-138 | H107, E110, R169, N178, R179, H235, G236, N242, D246, G298, Y302, V304, E324 | H107, E110, H235 |  | 1 |
| M14B | Scaffold00064.gene4781 Scaffold00064 | M14_AGBL5_like | [cd06236](http://www.ncbi.nlm.nih.gov/entrez/query.fcgi?cmd=Search&doptcmdl=GenPept&db=cdd&term=cd06236) | 2.64E-72 | H143, E146, R194, N203, R204 | H143, E146, |  |  |
|  |  | M14_AGBL5_like | [cd06236](http://www.ncbi.nlm.nih.gov/entrez/query.fcgi?cmd=Search&doptcmdl=GenPept&db=cdd&term=cd06236) | 2.88E-42 | D504, R505, H559, G560, C568M, E641 | H559 |  |  |
| M14B | Scaffold00229.gene8531 Scaffold00229 | M14_Nna1_like_2 | cd06235 | 1.94E-65 | H1096, E1099, R1147, N1156, R1157, H1194, G1195, V1204M | H1096, E1099, H1194 |  |  |
| M14B | Scaffold00271.gene8978 Scaffold00271 | M14_Nna1_like_2 | [cd06235](http://www.ncbi.nlm.nih.gov/entrez/query.fcgi?cmd=Search&doptcmdl=GenPept&db=cdd&term=cd06235) cl11393 | 5.22E-27 | H294, G295, I304M, E385 | H294 |  |  |
| M17 | LongOrf.asmbl_4972 Scaffold00014 | Peptidase_M17 | cd00433 | 8.15E-80 | K280, R355 | K268, D273, K280, D291, D351, E353, R354, L382 | 19, 20 |  |
| M17 | Scaffold00096.gene5884 Scaffold00096 | Peptidase_M17 | cd00433 | 4.00E-97 | K86, D91, K98, D109, D168, E170, R172, L197 | K86, D91, D109, D168, E170 |  |  |
| M17 | Scaffold00028.gene2941 Scaffold00028 | Peptidase_M17 | cd00433 | 4.20E-82 | K310, D315, K322, D333, D393, E395, R397, L425 | K310, D315, D333, D393, E395 |  |  |
| M17 | Scaffold00529.gene10048 Scaffold00529 | Peptidase_M17 | [cd00433](http://www.ncbi.nlm.nih.gov/entrez/query.fcgi?cmd=Search&doptcmdl=GenPept&db=cdd&term=cd00433) | 7.76E-19 | D7, D67, K69E | D7, D67, K69E |  |  |
| M18 | Scaffold00030.gene3073 Scaffold00030 | M18_DAP | cd05658 | 0 | H726, D891, N892, E925,E926, D999, Q1000M, H1002, K1027, Y1034, G1067, R1068T, M1092, H1093 | H726, D891, E925, E926, D999, H1093 |  |  |
|  |  | Peptidase_M18 | pfam02127 | 2.09E-157 |  |  |  |  |
| M20A | LongOrf.asmbl_20750 Scaffold00210 | M20_Aminoacylase-I like | [cd05646](http://www.ncbi.nlm.nih.gov/entrez/query.fcgi?cmd=Search&doptcmdl=GenPept&db=cdd&term=cd05646) | 1.90E-130 | D106, E171 | H105, D137, E171, E172, E226 |  |  |
| M20A | LongOrf.asmbl_20751 Scaffold00210 | M20_Aminoacylase-I like | [cd05646](http://www.ncbi.nlm.nih.gov/entrez/query.fcgi?cmd=Search&doptcmdl=GenPept&db=cdd&term=cd05646) | 7.48E-29 | - | H129 |  |  |
| M20 | LongOrf.asmbl_20752 Scaffold00210 | M20_Aminoacylase-I like | cd05646 | 2.69E-179 | D117, E182 | H115, D148, E182, E183, E237, H435 |  |  |
|  |  | Ac-peptdase-euk | TIGR01880 | 2.11E-154 |  |  |  |  |
| M20A | Scaffold00027.gene2904 Scaffold00027 | M20_dipept_like_CNDP | [cd05676](http://www.ncbi.nlm.nih.gov/entrez/query.fcgi?cmd=Search&doptcmdl=GenPept&db=cdd&term=cd05676) | 1.04E-129 | E26 E27, D55, H57L, L70T, R203, S240L, D274, A276S, T277, I278, H305 | E26 E27, D55, H305 |  |  |
| M20A | Scaffold00027.gene2905 Scaffold00027 | M20_dipept_like_CNDP | [cd05676](http://www.ncbi.nlm.nih.gov/entrez/query.fcgi?cmd=Search&doptcmdl=GenPept&db=cdd&term=cd05676) | 2.45E-31 | H53, D86, , | H53, D86, |  |  |
| M20A | Scaffold00210.gene8252 Scaffold00210 | M20_Aminoacylase-I like | [cd05646](http://www.ncbi.nlm.nih.gov/entrez/query.fcgi?cmd=Search&doptcmdl=GenPept&db=cdd&term=cd05646) | 8.75E-09 | - | H80 |  |  |
| M23B | LongOrf.asmbl_4837 Scaffold00014 | KISc_KHC_KIF5 | cd01369 | 7.50E-177 | R277, K280, R283 | R12, G84, A87S, G89, K90, T91, Y92H, D230 |  |  |
| M23B | LongOrf.asmbl_9815 Scaffold00043 | FYVE domain | cd00065 | 1.78E-15 | RKHHCR485-490, V495, R515 | C473, C476, C489, C492, C497, C500H, C516, C519 |  |  |
| M23B | LongOrf.asmbl_14099 Scaffold00086 | MYSc_type_II | cd01377 | 0 | ASSRFG235-240/IAGFEIFE458-465 | NPYKR122-12, Y127, P128, SGAGKTEN176-183 |  |  |
|  |  | MYSc | smart00242 | 0 | E1336H | Q1242H, L1246D, -1337H |  |  |
| M23B | LongOrf.asmbl_15932 Scaffold00110 | ABC_SMC1_euk | cd03275 | 1.62E-73 | GPNGSGKS41-49 | G46, S47, K49, S50, N51, Q147 (ATP binding site) |  |  |
|  |  | P-loop_NTPase | [cd03275](http://www.ncbi.nlm.nih.gov/entrez/query.fcgi?cmd=Search&doptcmdl=GenPept&db=cdd&term=cd03275) | 1.59E-52 |  | D1167, E1168, L1201 |  |  |
| M23B | LongOrf.asmbl_15526 Scaffold00104 | ABC_SMC3_euk | cd03272 | 3.62E-84 | GRNGSGKS43-50 | G46, S47, K49, S50, N51, Q152 | 19, 20 |  |
|  |  | ABC_SMC3_euk | cd03272 | 1.09E-52 | LSGGQKSLVA1125-1134 | D1154, E1155, F1185  ATP binding site |  |  |
| M23B | Scaffold00006.gene1018 Scaffold00006 | WD40 | cd00200 | 4.61E-31 | H352, T370S, D374, W380, N381D, E392V, F393H, V411C, D415, M420Y, N421, H475, D497, W503, D504, A536T, D540, E545, S557, E558H, V575A, E580D, Y585W, R586D, G601, H602, T620, D624, W629 |  |  |  |
| M23B | Scaffold00021.gene2393 Scaffold00021 | MYSc_type_II | cd01377 | 0 | NSSRFG223-228/IAGFEIFQ445-452, | NPYK100-103, L105, P106, SGAGKTEN153-160 |  |  |
|  |  | MYSc | smart00242 | 0 |  | S889H, A891H |  |  |
| M23B | Scaffold00004.gene697 Scaffold00004 | EGF_Lam | cd00055 | 1.01E-08 | C789, C791, C801, C808, C810, C819, EGF-like motif |  |  |  |
|  |  | EGF_Lam | cd00055 | 1.17E-08 |  |  |  |  |
|  |  | EGF_Lam | cd00055 | 1.80E-08 | C557, C559, C566, C573, C575, C584 EGF-like motif |  |  |  |
|  |  | EGF_Lam | cd00055 | 5.29E-08 |  |  |  |  |
|  |  | EGF_Lam | cd00055 | 1.19E-05 |  |  |  |  |
|  |  | EGF_Lam | cd00055 | 1.92E-05 |  |  |  |  |
|  |  | EGF_Lam | cd00055 | 0.000121 |  |  |  |  |
|  |  | SPEC | cd00176 | 0.000501 | A1222M, V1223E, R1224K, A1226L, K1227L, Q1227E | - |  |  |
| M24A | LongOrf.asmbl_8551 Scaffold00036 | Methionine Aminopeptidase 1 | cd01086 | 1.35E-111 | H188, D205, D216, H279, E312, E343 | - |  |  |
| M24 | LongOrf.asmbl_5871 Scaffold00019 | APP  (X-Prolyl Aminopeptidase 2) | cd01085 | 1.72E-80 | H451, D472, D483, A550S, E590, E604 |  | 20, 21 |  |
| M24A | LongOrf.asmbl_22742 Scaffold00312 | Methionine Aminopeptidase 1 | [cd01086](http://www.ncbi.nlm.nih.gov/entrez/query.fcgi?cmd=Search&doptcmdl=GenPept&db=cdd&term=cd01086) | 1.77E-46 | H121, D139, D150, H234, E257 | - |  |  |
| M24 | Scaffold00347.gene9486 Scaffold00347 | Prolidase | [cd01087](http://www.ncbi.nlm.nih.gov/entrez/query.fcgi?cmd=Search&doptcmdl=GenPept&db=cdd&term=cd01087) | 1.29e-23 | H286, D303, D314 | - |  |  |
| M24A | LongOrf.asmbl_1854 Scaffold00003 | Methionine Aminopeptidase 2 | cd01088 | 1.59E-159 | H224, D244, D255, H324, E357, E452 | - |  |  |
| M24B | LongOrf.asmbl_10238 Scaffold00047 | Prolidase | cd01087 | 5.83E-101 | H258, D279, D290, H373, E416, E456 | - |  |  |
| M24X | Scaffold00230.gene8539 Scaffold00230 | Related to aminopeptidase P and aminopeptidase M | [cd01091](http://www.ncbi.nlm.nih.gov/entrez/query.fcgi?cmd=Search&doptcmdl=GenPept&db=cdd&term=cd01091) | 1.50E-51 | N188, | S178D, N189D, F254H, N284E, K299E |  |  |
| M24X | LongOrf.asmbl_831 Scaffold00002 | PA2G4-like | cd01089 | 1.40E-64 | H111, D134, V145D, D223E | - |  |  |
| M28 | LongOrf.asmbl_2927 Scaffold00006 | M28_Fxna_like | cd03875 | 1.02E-97 | - | H182, D194, E228, E229, E255, H333 |  | 8 |
| M28A | LongOrf.asmbl_17290 Scaffold00130 | Zn-Peptidase Glutaminyl Cyclase | [cd03880](http://www.ncbi.nlm.nih.gov/Structure/cdd/cddsrv.cgi?uid=cd03880) | 8.32E-106 | E134D, E182 | D146, E183, H310 |  | 1 |
| M28B | LongOrf.asmbl_10476 Scaffold00049 | M28_PSMA_like [M28 Zn-peptidase prostate-specific membrane antigen | cd08022 | 3.03E-55 | H387, D397, E434, E435, G437, L438, D463, G524, Y556, H557 | H387, D397, E434, E435, D463, H557 |  |  |
| M28X | LongOrf.asmbl_1753 Scaffold00003 | Zn-Peptidase Nicalin, Nicastrin-like protein | cd03882 | 5.72E-57 | D235, G286E | H233, S249D, K287E, E320, | 23, 24 | 1 |
| M38 | Scaffold00157.gene7390 Scaffold00157 | D-HYD(D-hydantoinases) | cd01314 | 7.66E-21 | H153, L247K, S149H, P324H | - |  |  |
| M38 | LongOrf.asmbl_5355 Scaffold00016 | D-HYD(D-hydantoinases) | cd01314 | 0 | D349 , H91, H93, K183X, H216, H272 | - |  |  |
| M38 | Scaffold00016.gene1989 Scaffold00016 | D-HYD(D-hydantoinases) | cd01314 | 1.75E-86 | D64H, R66H, S156K, S212H, R252H, P195H, A349D | R266, G269 |  |  |
| M38 | Scaffold00011.gene1499 Scaffold00011 | Imidazolone-5PH | cd01296 | 3.48E-113 | H87, Y89H, H262, S265Q, H285, D336 | - |  |  |
| M41 | LongOrf.asmbl_11231 Scaffold00056 | AAA (ATPases Associated Activities) | cd00009 | 1.31E-26 |  | PPGTGKTL329-336, D387, N434A | 21, 22 | 2 |
|  | LongOrf.asmbl_8617 Scaffold00036 | AAA | cd00009 | 7.80E-11 |  | D390, N441 |  | 2 |
|  | LongOrf.asmbl_19571 Scaffold00176 | AAA | cd00009 | 2.21E-16 | R329 | T219, L220E, D270, N316A |  | 1 |
| M50A | LongOrf.asmbl_14141 Scaffold00087 | **S**2P-M50_PDZ_SREBP | [cd06162](http://www.ncbi.nlm.nih.gov/entrez/query.fcgi?cmd=Search&doptcmdl=GenPept&db=cdd&term=cd06162) | 4.46E-50 | E141, H140, H144 | E435N, L436A, F437I, S438P | 25, 26 | 6 |
|  |  | **S**2P-M50_PDZ_SREBP | [cd06162](http://www.ncbi.nlm.nih.gov/entrez/query.fcgi?cmd=Search&doptcmdl=GenPept&db=cdd&term=cd06162) | 1.57E-12 | N479, D487 | N479, V480, V481, P482 |  |  |
| M67A | LongOrf.asmbl_19705 Scaffold00180 | MPN_RPN11_CSN5 | cd08069 | 5.65E-140 | E54, H115, H117, S125, D128 | H115, H117, D128 |  |  |
| M67A | LongOrf.asmbl_1589 Scaffold00003 | MPN_RPN11_CSN5 | cd08069 | 5.91E-133 | E103, H165, H167, S175, D178, | H165, H167, D178 |  |  |
| M67C | LongOrf.asmbl_4529 Scaffold00013 | MPN_AMSH_like | cd08066 | 8.37E-89 | E241, H298, H300, S308, D311 | H298, H300, D311 |  |  |
|  | LongOrf.asmbl_20781 Scaffold00211 | Bin/Amphiphysin/Rvs domain of Sorting Nexins | [cd07596](http://www.ncbi.nlm.nih.gov/entrez/query.fcgi?cmd=Search&doptcmdl=GenPept&db=cdd&term=cd07596) | 9.77e-03 | S245H, S246, R248, E268, A271, L274 |  |  | 1 |

S4: serine proteases

| **Family** | **Gene ID** | **Serine proteases**  **Conserved domains** | **Domain family** | **E-Value** | **Active sites** | **Signal**  **sequence** | **TMHMN**  **helices** |
| --- | --- | --- | --- | --- | --- | --- | --- |
| S01A | LongOrf.asmbl_11010 Scaffold00053 | Trypsin-like serine protease | cd00190 | 3.77E-48 | H210, D278, S404 | 21 |  |
| S01A | Scaffold00011.gene1492 Scaffold00011 | Trypsin-like serine protease | cd00190 | 2.16E-57 | H92, D68, S271 | 22 |  |
| S01A | Scaffold00036.gene3378 Scaffold00036 | Trypsin-like serine protease | cd00190 | 1.42E-71 | H169, D229, S331, | 21 |  |
| S01A | Scaffold00158.gene7407 Scaffold00158 | Trypsin-like serine protease | cd00190 | 8.03E-46 | H194, D260, S351, |  | 1 |
| S01A | Scaffold00063.gene4723 Scaffold00063 | Trypsin-like serine protease | cd00190 | 4.59E-30 | H237, D310, T434S | 21 | 1 |
| S01A | Scaffold00025.gene2771 Scaffold00025 | LDLa | cd00112 | 4.96E-07 | D1752, D1756, D1762, E1763 |  | 1 |
|  |  | LDLa | cd00112 | 1.28E-06 | D1840, D1846, E1847 |  |  |
|  |  | LDLa | cd00112 | 0.003397 |  |  |  |
| S01A | Scaffold00005.gene846 Scaffold00005 | LDLa | cd00112 | 1.70E-08 | R867, D870, D874, D880, E881 | 30 | 1 |
|  |  | LDLa | cd00112 | 1.80E-08 | DGSDE:1275-12779 |  |  |
|  |  | LDLa | cd00112 | 1.25E-07 |  |  |  |
|  |  | LDLa | cd00112 | 1.78E-07 |  |  |  |
|  |  | LDLa | cd00112 | 2.83E-07 |  |  |  |
|  |  | LDLa | cd00112 | 3.58E-07 |  |  |  |
|  |  | LDLa | cd00112 | 6.79E-07 |  |  |  |
|  |  | LDLa | cd00112 | 8.82E-07 |  |  |  |
|  |  | LDLa | cd00112 | 2.13E-06 |  |  |  |
|  |  | LDLa | cd00112 | 2.38E-06 |  |  |  |
|  |  | LDLa | cd00112 | 2.97E-06 |  |  |  |
|  |  | LDLa | cd00112 | 3.13E-06 |  |  |  |
|  |  | LDLa | cd00112 | 3.33E-06 |  |  |  |
|  |  | LDLa | cd00112 | 4.64E-06 |  |  |  |
|  |  | LDLa | cd00112 | 4.70E-06 |  |  |  |
|  |  | LDLa | cd00112 | 1.22E-05 |  |  |  |
|  |  | LDLa | cd00112 | 8.65E-05 |  |  |  |
| S01B | Scaffold00038.gene3531 Scaffold00038 | PDZ_serine_protease (tryspin-like serine proteases) | cd00987 | 2.65E-09 | Y384E, L385, G386, L387V |  |  |
| S01B | LongOrf.asmbl_20696 Scaffold00209 | PDZ_signaling | cd00992 | 3.35E-20 | G203S, L204, G205, F206I, A259, V260, L263, R264 |  |  |
| S08A | Scaffold00039.gene3578 Scaffold00039 | Peptidases_S8_SKI-1_like | cd07479 | 1.98E-104 | D174, H219, F270, N290I, N319 | 22 | 1 |
| S08A | Scaffold00167.gene7569 Scaffold00167 | Peptidases_S8_Tripeptidyl_Aminopeptidase_II | [cd04857](http://www.ncbi.nlm.nih.gov/entrez/query.fcgi?cmd=Search&doptcmdl=GenPept&db=cdd&term=cd04857) | 6.70E-79 | H337, S575 |  |  |
| S08B | Scaffold00053.gene4314 Scaffold00053 | Peptidases_S8_Protein_convertases_Kexins_Furin-lik | cd04059 | 7.46E-85 | D15, D16, I64, H67, V100E, W118, R128Q |  |  |
| S08B | Scaffold00083.gene5386 Scaffold00083 | Peptidases_S8_Protein_convertases_Kexins_Furin-lik | cd04059 | 6.52E-149 | D38, D39, D75, H78, E121, W138, E148Q, N179, D190, S252 |  | 1 |
| S08B | Scaffold00211.gene8271 Scaffold00211 | Peptidases_S8_Protein_convertases_Kexins_Furin-lik | cd04059 | 6.93E-136 | D229, D230, F267D, H270, E313, W331, D341Q, D372N, D3812, S447 |  |  |
| S08B | Scaffold00006.gene937 Scaffold00006 | Peptidases_S8_Protein_convertases_Kexins_Furin-lik | cd04059 | 5.33E-164 |  |  | 1 |
| S08A | Scaffold00006.gene1002 Scaffold00006 | Cadherin_repeat | cd11304 | 2.87E-23 | E504, H505E, D564, E566, D595, N597, D598 |  | 1 |
|  |  | Cadherin_repeat | cd11304 | 2.97E-22 | Ca_2_^+^ binding site |  |  |
|  |  | Cadherin_repeat | cd11304 | 6.82E-21 |  |  |  |
|  |  | Cadherin_repeat | cd11304 | 1.36E-11 |  |  |  |
|  |  | Cadherin_repeat | cd11304 | 1.07E-07 |  |  |  |
| S08A | Scaffold00007.gene1037 Scaffold00007 | Cadherin_repeat | cd11304 | 4.88E-22 | E306, N307E, D362, E364, D397, N399, D400 |  | 2 |
|  |  | Cadherin_repeat | cd11304 | 7.00E-18 |  |  |  |
|  |  | Cadherin_repeat | cd11304 | 2.41E-10 |  |  |  |
|  |  | Cadherin_repeat | cd11304 | 2.24E-09 |  |  |  |
|  |  | Cadherin_repeat | cd11304 | 8.39E-09 |  |  |  |
| S08A | Scaffold00007.gene1092 Scaffold00007 | Cadherin_repeat | cd11304 | 2.11E-28 | E530, N531E, D480, E482, D513, N515, D516 |  |  |
|  |  | Cadherin_repeat | cd11304 | 2.56E-23 |  |  |  |
|  |  | Cadherin_repeat | cd11304 | 1.71E-16 |  |  |  |
|  |  | Cadherin_repeat | cd11304 | 1.97E-11 |  |  |  |
|  |  | Cadherin_repeat | cd11304 | 4.14E-06 |  |  | 1 |
| S08A | Scaffold00008.gene1266 Scaffold00008 | Cadherin_repeat | cd11304 | 4.57E-27 | E531, D532E, D589, E591, D622, N624, D625 | 17 | 1 |
|  |  | Cadherin_repeat | cd11304 | 7.02E-13 |  |  |  |
|  |  | Cadherin_repeat | cd11304 | 1.04E-12 |  |  |  |
|  |  | Cadherin_repeat | cd11304 | 4.90E-10 |  |  |  |
|  |  | Cadherin_repeat | cd11304 | 1.14E-08 |  |  |  |
|  |  | Cadherin_repeat | cd11304 | 1.90E-07 |  |  |  |
|  |  | Cadherin_repeat | cd11304 | 4.80E-05 |  |  |  |
| S08A | Scaffold00009.gene1307 Scaffold00009 | Cadherin_repeat | cd11304 | 3.00E-23 | E285, N286E, D345, E347, D380, N382, D383 | 21 | 1 |
|  |  | Cadherin_repeat | cd11304 | 4.87E-20 |  |  |  |
|  |  | Cadherin_repeat | cd11304 | 9.68E-12 |  |  |  |
|  |  | Cadherin_repeat | cd11304 | 1.98E-06 |  |  |  |
|  |  | Cadherin_repeat | cd11304 | 6.92E-05 |  |  |  |
| S08A | Scaffold00038.gene3515 Scaffold00038 | Cadherin_repeat | cd11304 | 8.53E-30 | E315, S316E, D371, E373, D404, N406, D407, | 26 |  |
|  |  | Cadherin_repeat | cd11304 | 2.70E-28 | Ca^2+^ binding site |  |  |
|  |  | Cadherin_repeat | cd11304 | 2.54E-17 |  |  |  |
|  |  | Cadherin_repeat | cd11304 | 5.04E-13 |  |  |  |
|  |  | Cadherin_repeat | cd11304 | 1.40E-06 |  |  |  |
| S08A | Scaffold00003.gene565 Scaffold00003 | Cadherin_repeat | cd11304 | 2.18E-29 |  |  | 1 |
|  |  | Cadherin_repeat | cd11304 | 3.33E-25 |  |  |  |
|  |  | Cadherin_repeat | cd11304 | 6.03E-24 |  |  |  |
|  |  | Cadherin_repeat | cd11304 | 6.42E-23 |  |  |  |
|  |  | Cadherin_repeat | cd11304 | 2.04E-20 |  |  |  |
|  |  | Cadherin_repeat | cd11304 | 8.53E-19 |  |  |  |
|  |  | Cadherin_repeat | cd11304 | 8.55E-19 |  |  |  |
|  |  | Cadherin_repeat | cd11304 | 5.74E-18 |  |  |  |
|  |  | Cadherin_repeat | cd11304 | 7.54E-14 |  |  |  |
|  |  | Cadherin_repeat | cd11304 | 4.07E-13 |  |  |  |
|  |  | Cadherin_repeat | cd11304 | 1.29E-12 |  |  |  |
|  |  | Cadherin_repeat | cd11304 | 1.64E-12 |  |  |  |
|  |  | Cadherin_repeat | cd11304 | 2.16E-12 |  |  |  |
|  |  | Cadherin_repeat | cd11304 | 5.08E-12 |  |  |  |
|  |  | Cadherin_repeat | cd11304 | 7.73E-11 |  |  |  |
|  |  | Cadherin_repeat | cd11304 | 9.50E-08 |  |  |  |
|  |  | Cadherin_repeat | cd11304 | 1.16E-07 |  |  |  |
|  |  | Cadherin_repeat | cd11304 | 1.32E-07 |  |  |  |
|  |  | Cadherin_repeat | cd11304 | 2.15E-07 |  |  |  |
|  |  | Cadherin_repeat | cd11304 | 3.36E-06 |  |  |  |
|  |  | Cadherin_repeat | cd11304 | 4.78E-06 |  |  |  |
|  |  | Cadherin_repeat | cd11304 | 1.18E-05 |  |  |  |
|  |  | Cadherin_repeat | cd11304 | 4.72E-05 |  |  |  |
|  |  | Cadherin_repeat | cd11304 | 5.18E-05 |  |  |  |
|  |  | Cadherin_repeat | cd11304 | 0.000646 |  |  |  |
|  |  | Cadherin_repeat | cd11304 | 0.00149 |  |  |  |
|  |  | EGF_CA | cd00054 | 3.24E-07 |  |  |  |
|  |  | EGF_CA | cd00054 | 2.82E-05 |  |  |  |
|  |  | EGF_CA | cd00054 | 0.000864 |  |  |  |
| S09C | Scaffold00028.gene2995 Scaffold00028 | Peptidase_S9 | pfam00326 | 2.60E-19 |  |  |  |
|  |  | Esterase_lipase | cd00312 | 2.60E-19 |  |  |  |
|  |  | DAP2 | COG1506 | 3.39E-37 |  |  |  |
|  |  | KU70 | cd00788 | 3.16E-41 |  |  |  |
|  |  | vWA_ku | cd01458 | 2.71E-26 |  |  |  |
| S09X | Scaffold00009.gene1314 Scaffold00009 | Esterase_lipase | cd00312 | 2.67E-27 | G317S, E458 |  | 1 |
| S09X | Scaffold00028.gene2975 Scaffold00028 | Esterase_lipase | cd00312 | 1.02E-34 | S154, A192E, Y386H |  | 1 |
| S09X | Scaffold00107.gene6179 Scaffold00107 | Esterase_lipase | [cd00312](http://www.ncbi.nlm.nih.gov/entrez/query.fcgi?cmd=Search&doptcmdl=GenPept&db=cdd&term=cd00312) | 4.05E-104 | S225, E383, H527 | 18 |  |
| S09X | Scaffold00265.gene8929 Scaffold00265 | Esterase_lipase | cd00312 | 4.20E-111 | G266S, E421, Q553H | 26 |  |
| S09X | Scaffold00002.gene282 Scaffold00002 | Esterase_lipase | cd00312 | 1.58E-19 | D332S |  | 1 |
| S09X | Scaffold00093.gene5793 Scaffold00093 | Esterase_lipase | [cd00312](http://www.ncbi.nlm.nih.gov/entrez/query.fcgi?cmd=Search&doptcmdl=GenPept&db=cdd&term=cd00312) | 2.15E-49 | G278S, E406, H523 |  | 1 |
| S09X | LongOrf.asmbl_9807 Scaffold00043 | Esterase_lipase | [cd00312](http://www.ncbi.nlm.nih.gov/entrez/query.fcgi?cmd=Search&doptcmdl=GenPept&db=cdd&term=cd00312) | 6.87E-122 | S242, E371, H509 | 20 |  |
| S14 | LongOrf.asmbl_592 Scaffold00001 | S14_ClpP_2 | cd07017 | 9.85E-107 | S134, H159, D208 |  |  |
| S16 | LongOrf.asmbl_17867 Scaffold00139 | AAA | cd00009 | 1.45E-14 | P555-S562, D621, | 18 |  |
| S26 | LongOrf.asmbl_13939 Scaffold00084 | S26_SPase_I | cd06530 | 1.32E-19 | S79, H119K |  | 2 |
| S26A | Scaffold00011.gene1457 Scaffold00011 | S26_SPase_I | cd06530 | 1.27E-08 | K27 |  |  |
| S41A | LongOrf.asmbl_15758 Scaffold00107 | PDZ_signaling | cd00992 | 1.03E-24 | L100M, G101, V104IL |  |  |
| S41A | LongOrf.asmbl_20696 Scaffold00209 | PDZ_signaling | cd00992 | 5.57E-16 |  |  |  |
| S41A | LongOrf.asmbl_2208 Scaffold00004 | PDZ_signaling | cd00992 | 6.90E-14 | G118, L119I, L121V, A166L, I170L, K171R, |  |  |
|  |  | PDZ_signaling | cd00992 | 1.99E-10 | S200, L201, G202, I203, V205, I246A, R247, I250L, K251 |  |  |
| S41A | LongOrf.asmbl_4928 Scaffold00014 | PDZ_signaling | cd00992 | 1.94E-14 | G14S, L15M, G16, L17I, I19V, V69A, L70V, L73, R74 |  |  |
|  |  | PDZ_signaling | cd00992 | 1.33E-13 | G373S, T374L, G375, I376, I378L, A424, A425 |  |  |
|  |  | PDZ_signaling | cd00992 | 1.07E-09 | L189M, G190, L193V, A234L, R239 |  |  |
| S41A | Scaffold00016.gene1949 Scaffold00016 | PDZ_signaling | cd00992 | 5.04E-16 | G495S, Y496L, G497L, L498I, V500, V544A, V545, I548, R549 |  |  |
| S41A | Scaffold00212.gene8295 Scaffold00212 | PDZ_signaling | cd00992 | 9.75E-16 | S1072, L1073, G1074, L1075I, I1077V, A1033, V1034, L1037, M1038R |  |  |
|  |  | PDZ_signaling | cd00992 | 1.91E-15 |  |  |  |
|  |  | PDZ_signaling | cd00992 | 3.32E-11 |  |  |  |
|  |  | PDZ_signaling | cd00992 | 2.68E-06 |  |  |  |
| S54 | Scaffold00058.gene4515 Scaffold00058 | Rhomboid | pfam01694 | 1.01E-12 | no |  | 6 |
|  |  | EFh | cd00051 | 0.00117 | D43, E52, D77, N79D, D81, Q88E |  |  |

| **Family** | **Gene ID** | **T****hreonine proteases**  **Conserved domains** | **Domain**  **family** | **E-Value** | **Active sites** | **Signal**  **sequence** | **TMHMN**  **helices** |
| --- | --- | --- | --- | --- | --- | --- | --- |
| T01A | LongOrf.asmbl_14062 Scaffold00085 | proteasome_alpha_type_1 | cd03749 | 4.15185e-120 | S33T, R51K, K62, S163A |  |  |
|  | LongOrf.asmbl_19945 Scaffold00188 | proteasome_alpha_type_2 | cd03750 | 1.70591e-129 | A47G, E63V, K65, K81 |  |  |
|  | LongOrf.asmbl_6563 Scaffold00022 | proteasome_alpha_type_3 | cd03751 | 1.25428e-120 | G35S, E51D, I53L, R66, K167 |  |  |
|  | Scaffold00019.gene2216 Scaffold00019 | proteasome_alpha_type_4 | [cd03752](http://www.ncbi.nlm.nih.gov/entrez/query.fcgi?cmd=Search&doptcmdl=GenPept&db=cdd&term=cd03752) | 1.15398e-104 | E43, R45, K59 |  |  |
|  | Scaffold00156.gene7357 Scaffold00156 | proteasome_alpha_type_5 | cd03753 | 3.56307e-95 | E200, R202, K215, S320 |  |  |
|  | LongOrf.asmbl_8862 Scaffold00037 | proteasome_alpha_type_6 | cd03754 | 1.53686e-118 | L60, Q76, K78, R91H | 25 |  |
|  | LongOrf.asmbl_5775 Scaffold00018 | proteasome_alpha_type_7 | cd03755 | 3.50892e-118 | S32, E48, K50, K63 |  |  |
|  | LongOrf.asmbl_17280 Scaffold00130 | proteasome_beta_type_1 | cd03757 | 2.77494e-85 | G11, D27, R29, H45K |  |  |
|  | LongOrf.asmbl_6442 Scaffold00021 | proteasome_beta_type_2 | cd03758 | 1.74708e-100 | —— |  |  |
|  | LongOrf.asmbl_16068 Scaffold00112 | proteasome_beta_type_3 | cd03759 | 3.19074e-104 | S9G, D25, R27, K41, S141T, D178, S181, G182 |  |  |
|  | LongOrf.asmbl_17241 Scaffold00129 | proteasome_beta_type_4 | cd03760 | 6.38916e-93 | T28, T44, L46G, R60, Y161F, D205, A208S |  |  |
|  | LongOrf.asmbl_21090 Scaffold00222 | proteasome_beta_type_5 | cd03761 | 9.30661e-113 | T89, D105, R107, K121R, S217, D255, S258, G259 |  |  |
|  | LongOrf.asmbl_251 Scaffold00001 | proteasome_beta_type_6 | cd03762 | 4.87116e-103 | T41, D57, R59, K73, S170, D207, S210, G211 |  |  |
|  | LongOrf.asmbl_11803 Scaffold00062 | proteasome_beta_type_7 | cd03763 | 3.99903e-110 | T39, D55, R57, K71, S166,D204, S207, G208 |  |  |
| T02 | LongOrf.asmbl_11603 Scaffold00060 | Glycosylasparaginase | cd04513 | 1.30174e-97 | W42, HDT216-218, T236, R246, T269 | 19 |  |
|  | Scaffold00003.gene529 Scaffold00003 | Taspase1_like | cd04514 | 1.6722e-73 | A8, G9, T62, N63, T196, V197 |  |  |

S5: threonine proteases
