# Supplementary material for: Genome-wide analysis of regulatory proteases sequences identified through bioinformatics data mining in Taenia solium
Source: BMC Genomics. 2014 Jun 4;15:428. doi: 10.1186/1471-2164-15-428 (PMC4070553; doi:10.1186/1471-2164-15-428)
Supplement: Supplementary file 2 — Additional file 2: KAAS analysis: KEGG pathway assignment and KEGG orthology number (KO number) for Taenia solium proteases. Bioinformatic analysis using the Kyoto Encyclopedia of Genes and Genomes used to predict probable functions and the cellular processes for the tapeworm proteases, based on orthologous relationships of proteases for which functions in other species have been clearly established. (DOC 58 KB) [file 12864_2013_6143_MOESM2_ESM.doc]

**Results of KAAS analysis: The KEGG pathway assignment and KEGG orthology number (KO number) of each *T. solium* protease.**

01100 Metabolism

**01101** **Carbohydrate Metabolism**

ko00520 Amino sugar and nucleotide sugar metabolism (1)

**01102 Energy Metabolism**

ko00910 Nitrogen metabolism (1)

**01103 Lipid Metabolism**

ko00564 Glycerophospholipid metabolism (1)

ko00590 Arachidonic acid metabolism (1)

**01104 Nucleotide Metabolism**

ko00240 Pyrimidine metabolism (1)

**01105 Amino Acid Metabolism**

ko00250 Alanine, aspartate and glutamate metabolism (2)

ko00330 Arginine and proline metabolism (2)

ko00340 Histidine metabolism (2)

**01106 Metabolism of Other Amino Acids**

ko00410 beta-Alanine metabolism (2)

ko00480 Glutathione metabolism (2)

**01107 Glycan Biosynthesis and Metabolism**

ko00511 Other glycan degradation (1)

**01109 Metabolism of Cofactors and Vitamins**

ko00770 Pantothenate and CoA biosynthesis (1)

**ko****01110** Biosynthesis of secondary metabolites (3)

**01111 Xenobiotics Biodegradation and Metabolism**

ko00983 Drug metabolism - other enzymes (1)

**1.12 Reaction module maps**

ko01210 2-Oxocarboxylic acid metabolism (2)

ko01120 Microbial metabolism in diverse environments (1)

01120 Genetic Information Processing

**01121 Transcription**

ko03040 Spliceosome (1)

**01123 Folding, Sorting and Degradation**

ko03060 Protein export (2)

ko04141 Protein processing in endoplasmic reticulum (2)

ko03050 Proteasome (14)

01130 Environmental Information Processing

**01132 Signal Transduction**

ko04010 MAPK signaling pathway (1)

ko04310 Wnt signaling pathway (1)

ko04330 Notch signaling pathway (1)

ko04340 Hedgehog signaling pathway (1)

ko04390 Hippo signaling pathway (1)

ko04391 Hippo signaling pathway - fly (1)

ko04151 PI3K-Akt signaling pathway (1)

**01133 Signaling Molecules and Interaction**

ko04512 ECM-receptor interaction (1)

01140 Cellular Processes

**01141 Transport and catabolism**

ko04144 Endocytosis (2)

ko04145 Phagosome (2)

ko04142 Lysosome (4)

**01143 Cell Growth and Death**

ko04110 Cell cycle (2)

ko04111 Cell cycle - yeast (2)

ko04112 Cell cycle - Caulobacter (1)

ko04113 Meiosis - yeast (2)

ko04114 Oocyte meiosis (2)

ko04210 Apoptosis (1)

ko04115 p53 signaling pathway (1)

**01144 Cell Communication**

ko04510 Focal adhesion (1)

ko04530 Tight junction (1)

01150 Organismal Systems

**01151 Immune System**

ko04640 Hematopoietic cell lineage (1)

ko04650 Natural killer cell mediated cytotoxicity (1)

ko04612 Antigen processing and presentation (2)

**01152 Endocrine system**

ko04614 Renin-angiotensin system (1)

**01156 Nervous system**

ko04725 Cholinergic synapse (1)

ko04728 Dopaminergic synapse (1)

ko04726 Serotonergic synapse (1)

01160 Human Diseases

**01161 Cancers**

ko05200 Pathways in cancer (2)

ko05202 Transcriptional misregulation in cancer (1)

ko05203 Viral carcinogenesis (3)

ko05210 Colorectal cancer (1)

ko05222 Small cell lung cancer (1)

**01162 Immune diseases**

ko05323 Rheumatoid arthritis (1)

**01163 Neurodegenerative Diseases**

ko05010 Alzheimer's disease (3)

ko05012 Parkinson's disease (1)

ko05014 Amyotrophic lateral sclerosis (ALS) (1)

ko05016 Huntington's disease (1)

ko05020 Prion diseases (1)

**01164 Cardiovascular diseases**

ko05416 Viral myocarditis (2)

**01165 Endocrine and Metabolic Disorders**

ko04940 Type I diabetes mellitus (1)

**01166 Infectious Diseases**

ko05120 Epithelial cell signaling in Helicobacter pylori infection (3)

ko05133 Pertussis (1)

ko05134 Legionellosis (1)

ko05152 Tuberculosis (3)

ko05164 Influenza A (1)

ko05161 Hepatitis B (1)

ko05168 Herpes simplex infection (2)

ko05169 Epstein-Barr virus infection (2)

ko05146 Amoebiasis (2)

ko05145 Toxoplasmosis (2)

Numbers, protease and orthology

1. LongOrf.asmbl_10039 K01379
2. Scaffold00013.gene1644
3. Scaffold00024.gene2637
4. Scaffold00053.gene4324
5. Scaffold00010.gene1446
6. Scaffold00013.gene1691
7. Scaffold00116.gene6445
8. Scaffold00136.gene6963
9. Scaffold00155.gene7338
10. Scaffold00222.gene8405
11. Scaffold00293.gene9160
12. Scaffold00443.gene9836
13. Scaffold00607.gene10168
14. Scaffold00609.gene10171
15. Scaffold01070.gene10611
16. Scaffold01299.gene10720
17. Scaffold01695.gene10847
18. Scaffold03691.gene11201
19. Scaffold00287.gene9105
20. Scaffold00001.gene83 K07497
21. Scaffold00071.gene5019 K07497
22. LongOrf.asmbl_1043 K01363
23. Scaffold00002.gene342
24. LongOrf.asmbl_24428
25. LongOrf.asmbl_6319 K15621
26. Scaffold00212.gene8293
27. Scaffold00115.gene6434 K01365
28. LongOrf.asmbl_24242
29. Scaffold00009.gene1353
30. LongOrf.asmbl_4585
31. LongOrf.asmbl_13767 K08576
32. LongOrf.asmbl_13200
33. Scaffold00130.gene6821
34. Scaffold00049.gene4123
35. Scaffold00078.gene5230
36. LongOrf.asmbl_4936 K05609
37. LongOrf.asmbl_7418 K05610
38. Scaffold00017.gene2074 K16576
39. LongOrf.asmbl_3186
40. Scaffold00004.gene651 K02187
41. Scaffold00079.gene5290
42. LongOrf.asmbl_16154
43. Scaffold01059.gene10604
44. Scaffold01059.gene10605
45. Scaffold00125.gene6704 K01304
46. LongOrf.asmbl_21266 K11851
47. LongOrf.asmbl_20169 K11841
48. LongOrf.asmbl_8072
49. Scaffold00054.gene4346 K11852
50. Scaffold00156.gene7356
51. LongOrf.asmbl_23107 K11855
52. LongOrf.asmbl_1417 K11835
53. LongOrf.asmbl_23799 K11843
54. LongOrf.asmbl_23911
55. LongOrf.asmbl_24548 K11366
56. Scaffold00061.gene4636
57. Scaffold00051.gene4220 K11836
58. Scaffold00037.gene3444 K11838
59. Scaffold00034.gene3256 K11840
60. LongOrf.asmbl_3312 K11838
61. LongOrf.asmbl_11082 K11842
62. LongOrf.asmbl_8663 K11844
63. LongOrf.asmbl_4731 K11858
64. LongOrf.asmbl_3982 K12847
65. Scaffold00186.gene7880 K11833
66. LongOrf.asmbl_11541 K11848
67. Scaffold00051.gene4200 K00820
68. Scaffold00099.gene5950 K00264
69. Scaffold00067.gene4884 K11989
70. LongOrf.asmbl_7288 K03152
71. LongOrf.asmbl_13755
72. LongOrf.asmbl_23061 K08776
73. Scaffold00065.gene4802
74. Scaffold00104.gene6118 K11140
75. Scaffold00263.gene8897
76. Scaffold00263.gene8901
77. Scaffold00346.gene9478
78. Scaffold00568.gene10114
79. Scaffold00806.gene10404
80. Scaffold00045.gene3907 K01254
81. Scaffold00004.gene624
82. LongOrf.asmbl_7835 K01410
83. Scaffold00054.gene4368
84. LongOrf.asmbl_12456
85. LongOrf.asmbl_2871 K06059
86. Scaffold00006.gene972
87. Scaffold00064.gene4768 K06704
88. Scaffold00011.gene1489
89. Scaffold00003.gene496
90. Scaffold00168.gene7594
91. Scaffold00082.gene5370
92. Scaffold00122.gene6610 K01415
93. LongOrf.asmbl_22677
94. LongOrf.asmbl_9553 K01294
95. Scaffold00064.gene4781
96. Scaffold00229.gene8531
97. Scaffold00271.gene8978
98. LongOrf.asmbl_4972 K01255
99. Scaffold00096.gene5884 K01255
100. Scaffold00028.gene2941 K01255
101. Scaffold00529.gene10048
102. Scaffold00030.gene3073 K01267
103. LongOrf.asmbl_20750 K01293
104. LongOrf.asmbl_20751
105. LongOrf.asmbl_20752 K14677
106. Scaffold00027.gene2904 K08660
107. Scaffold00027.gene2905
108. Scaffold00210.gene8252
109. LongOrf.asmbl_4837 K10396
110. LongOrf.asmbl_9815 K12478
111. LongOrf.asmbl_14099 K10352
112. LongOrf.asmbl_15932 K06636
113. LongOrf.asmbl_15526 K06669
114. Scaffold00006.gene1018
115. Scaffold00021.gene2393 K10352
116. Scaffold00004.gene697 K05635
117. LongOrf.asmbl_8551 K01265
118. LongOrf.asmbl_5871 K01262
119. LongOrf.asmbl_22742
120. Scaffold00347.gene9486 K01262
121. LongOrf.asmbl_1854 K01265
122. LongOrf.asmbl_10238 K14213
123. Scaffold00230.gene8539
124. LongOrf.asmbl_831 K14813
125. LongOrf.asmbl_2927
126. LongOrf.asmbl_17290 K00683
127. LongOrf.asmbl_10476
128. LongOrf.asmbl_1753
129. Scaffold00157.gene7390
130. LongOrf.asmbl_5355 K01464
131. Scaffold00016.gene1989
132. Scaffold00011.gene1499 K01468
133. LongOrf.asmbl_11231 K08956
134. LongOrf.asmbl_8617 K09552
135. LongOrf.asmbl_19571 K08955
136. LongOrf.asmbl_14141 K07765
137. LongOrf.asmbl_19705 K03030
138. LongOrf.asmbl_1589 K09613
139. LongOrf.asmbl_4529 K11866
140. LongOrf.asmbl_20781
141. LongOrf.asmbl_11010
142. Scaffold00011.gene1492
143. Scaffold00036.gene3378 K09634
144. Scaffold00158.gene7407
145. Scaffold00063.gene4723
146. Scaffold00025.gene2771 K03068
147. Scaffold00005.gene846
148. Scaffold00038.gene3531 K01362
149. LongOrf.asmbl_20696
150. Scaffold00039.gene3578 K08653
151. Scaffold00167.gene7569 K01280
152. Scaffold00053.gene4314
153. Scaffold00083.gene5386
154. Scaffold00211.gene8271 K01360
155. Scaffold00006.gene937 K01349
156. Scaffold00006.gene1002
157. Scaffold00007.gene1037
158. Scaffold00007.gene1092
159. Scaffold00008.gene1266
160. Scaffold00009.gene1307
161. Scaffold00038.gene3515
162. Scaffold00003.gene565
163. Scaffold00028.gene2995 K01303
164. Scaffold00009.gene1314
165. Scaffold00028.gene2975
166. Scaffold00107.gene6179
167. Scaffold00265.gene8929
168. Scaffold00002.gene282
169. Scaffold00093.gene5793
170. LongOrf.asmbl_9807 K01049
171. LongOrf.asmbl_592 K01358
172. LongOrf.asmbl_17867 K08675
173. LongOrf.asmbl_13939 K13280
174. Scaffold00011.gene1457 K09647
175. LongOrf.asmbl_15758
176. LongOrf.asmbl_20696
177. LongOrf.asmbl_2208
178. LongOrf.asmbl_4928
179. Scaffold00016.gene1949
180. Scaffold00212.gene8295 K16175
181. Scaffold00058.gene4515 K02857
182. LongOrf.asmbl_14062 K02725
183. LongOrf.asmbl_19945 K02726
184. LongOrf.asmbl_6563 K02727
185. Scaffold00019.gene2216 K02728
186. Scaffold00156.gene7357 K02729
187. LongOrf.asmbl_8862 K02730
188. LongOrf.asmbl_5775 K02731
189. LongOrf.asmbl_17280 K02732
190. LongOrf.asmbl_16068 K02735
191. LongOrf.asmbl_17241 K02736
192. LongOrf.asmbl_21090 K02737
193. LongOrf.asmbl_251 K02738
194. LongOrf.asmbl_11803 K02739
195. LongOrf.asmbl_11603 K01444
196. Scaffold00003.gene529 K08657
