# Supplementary material for: Genome-wide analysis of regulatory proteases sequences identified through bioinformatics data mining in Taenia solium
Source: BMC Genomics. 2014 Jun 4;15:428. doi: 10.1186/1471-2164-15-428 (PMC4070553; doi:10.1186/1471-2164-15-428)
Supplement: Supplementary file 3 — Additional file 3: C1_S1 family catalytic residues - active sites shown in black of blue. Partial sequence alignment of a family of proteases for several species; active site residues central to catalysis are highlighted. (DOC 78 KB) [file 12864_2013_6143_MOESM3_ESM.doc]

Figure S1: C1 family (Cathepsin) catalytic motif (active site shown in black)

| Human_CATO[P43234]  Mouse_CATO[Q8BM88]  Human_CATFF[Q9UBX1]  Mouse_CATF[Q9R013]  Human_CATW[P56202]  Mouse_CATW[P56203]  Human_CATH[P09668]  Mouse_CATH[P49935] *T.solium*[LongOrf.asmbl_6319]  *T.solium*[LongOrf.asmbl_24242]  *T.solium*[Scaffold00115.gene6434]  *E.multilocularis*_CATL[Q0WYD8]  *F.hepatica*_CATLL[Q24940]  *T.asiatica*_CATL[B7XBA1]  *T.solium*[LongOrf.asmbl_24428]  *T.saginata*_CATL[B7XBA0]  T.pisiformis_CESTL[F6MEN8]  *T.solium*[Scaffold00212.gene8293]  Human_CATK[P43235]  Mouse_CATK[P55097]  Human_CATS[P25774]  Mouse_CATS[NP_001254624]  Human_CATL[P07711] Human_CATV[O60911]  Mouse_CATL[P06797]  Mouse_CATM[Q9JL96]  Mouse_Cts3[Q9DAZ8]  Mouse_Cts2[Q9JI81]  Mouse_CATR[Q9JIA9]  Mouse_Cts6[Q9ET52]  Mouse_CATP[Q9R014]  Mouse_Cts1[Q9JI84  Fruit fly_CATL[Q95029]  *T.solium*[Scaffold00009.gene1353]  *T.solium*[LongOrf.asmbl_1043]  *E.multilocularis*_CATL[E9RH13]  S.mansoni_CB2[Q95PM1]  S.japonicum_CB[Q7Z1I6]  T.regenti_CB2A7[A7L844]  Human_CATB[P07858]  Mouse_CATB[P10605]  *T.solium*[Scaffold00002.gene342]  Fruit fly_CATB[NP_001259536]  *S.mansoni*_CYSP[P25792]  *S.japonicum*_CATB[P43157]  *C.elegans*_CPR6[P43510]  *C.elegans*_CPR3[P43507]  *C.elegans*_CPR6[Q8MQC6]  *C.elegans*_CPR5[P43509]  Human_CATZ[Q9UBR2]  Mouse_CATZ[Q9WUU7]  *C.elegans*_CPZ1[G5EGP8] | VVTQVRNQQMCGGCWAFSVVGA 115  VVNPVRNQEMCGGCWAFSVVSA 114  AVTKVKDQGMCGSCWAFSVTGN 122  AVTEVKNQGMCGSCWAFSVTGN 122  AISPIKDQKNCNCCWAMAAAGN 125  IISSVKNQGSCKCCWAMAAADN 125  FVSPVKNQGACGSCWTFSTTGA 120  VVSPVKNQGACASCWTFSTTGA 120  RVTGVKDQGHCGSCWAFSAIGA 124  --------------WAFSATGA 8  LVTPIKDQGPCGSCWAFSATGA 130  LVTPIKDQGDCGSCWAFSATGA 130  YVTEVKDQGNCGSCWAFSTTGT 123  LVTEVKNQGNCGSCWAFSSTGA 127  LVTEVKNQGNCGSCWAFSSTGA 127  LVTEVKNQGNCGSCWAFSSTGA 127  LVTEVKNQGNCGSCWAFSSTGA 127  ----------------------  YVTPVKNQGQCGSCWAFSSVGA 127  YVTPVKNQGQCGSCWAFSSAGA 127  CVTEVKYQGSCGACWAFSAVGA 125  CVTEVKYQGSCGACWAFSAVGA 125  YVTPVKNQGQCGSCWAFSATGA 123  YVTPVKNQKQCGSCWAFSATGA 123  CVTPVKNQGQCGSCWAFSASGC 123  YVTPVQTQGRCNSCWAFSVTGA 123  YVTPARTQIACNSCWAISVTGA 123  CVTPVKNQGTCNSCWAFSAAGA 123  YVTPVRRQGDCDACWAFAVTGA 124  YVTRVRRQKFCNSCWAFAVNGA 124  YVTPVRNQGKCGSCWAFAAAGA 123  YVTPVRRQGSCGACWAFSVTAC 121  AVTAVKDQGHCGSCWAFSSTGA 133  PNRKVRDQLSCGSCWAFASVSA 134  TLFEIRDQGSCGSCWAFGAAEA 101  TLFEIRDQGSCGSCWAFGAAEA 100  SISEIRDQSSCGSCWAFGAVEA 100  SISEIRDQSSCGSCWAFGAVEA 102  SISEIRDQSSCGSCWAFGAVEA 102  TIKEIRDQGSCGSCWAFGAVEA 97  TIGQIRDQGSCGSCWAFGAVEA 97  TIGEIRDQGTCGSCWAFGATEA 106  TIGEIRDQGSCGSCWAFGAVEA 96  SIATIRDQSRCGSCWSFGAVEA 102  SISQIRDQSRCGSCWAFGAVEA 102  SIKVIRDQSSCGSCWAFGAVEA 106  TIKLIRNQATCGSCWAFGAAEV 102  SIKLIRDQATCGSCWAFGAAEM 97  SINNIRDQSDCGSCWAFAAAEA 94  ITRNQHIPQYCGSCWAHASTSA 76  VTRNQHIPQYCGSCWAHGSTSA 76  ADRNQHIPQYCGSCWAFGATSA 80 | ANHAVLITGFDK--------------------TGSTPYWIVRNSWGSS  ANHAVLITGFDR--------------------TGNTPYWMVRNSWGSS  IDHAVLLVGYG--------------NRS------DVPFWAIKNSWGTD  IDHAVLLVGYG--------------NRS------NIPYWAIKNSWGSD  VDHSVLLVGFGSVKSEEGIWAETVSSQSQPQPPHPTPYWILKNSWGAQ  VDHSVLLVGFG--KKKEGMQTGTVLSHSRKRR-HSSPYWILKNSWGAH  VNHAVLAVGYG--------------------EKNGIPYWIVKNSWGPQ  VNHAVLAVGYG--------------------EQNGLLYWIVKNSWGSQ  LNHGVLLAGYG--------------------EQNGIPYWLIKNSWGTN  LDHGVLAVGYD-------------------ADKARRNYWIVKNSWGKQ  LDHGVLVVGYN-------------------ADKTRQKYWIVKNSWGEQ  LDHAVLVVGYD-------------------ADKTRQKYWIVKNSWGED  VNHAVLAVGYG--------------------TQGGTDYWIVKNSWGTY  LNHGVLAIGYG--------------------KQEGKPYWLVKNSWGTR  ------------------------------------------------  LNHGVLAIGYG--------------------KQDGKPYWLVKNSWGTR  LNHGVLAVGYG--------------------KLDGKPYWLVKNSWGSG  LNHGVLAIGYG--------------------KQDGKPYWLVKNSWGTR  LNHAVLAVGYG--------------------IQKGNKHWIIKNSWGEN  VNHAVLVVGYG--------------------TQKGSKHWIIKNSWGES  VNHGVLVVGYG--------------------DLNGKEYWLVKNSWGHN  VNHGVLVVGYG--------------------TLDGKDYWLVKNSWGLN  MDHGVLVVGYGF----------------ESTESDNNKYWLVKNSWGEE  LDHGVLVVGYGF----------------EGANSNNSKYWLVKNSWGPE  LDHGVLLVGYGY----------------EGTDSNKNKYWLVKNSWGSE  VTHSMLLVGYGF----------------TGRESDGRKYWLVKNSMGTQ  LRHAVLLVGYGF----------------IGRESEGRKYWIIKNSLGTK  INHSVLVVGYGY----------------EGKESDGNKYWLIKNSHGEQ  VTHGVLVVGYGF----------------KGIETDGNHYWLIKNSWGKR  VNHAVLVVGYGT----------------EGNETDGNKYWLIKNSWGRR  VNHAVLVVGYGS----------------EGDVKDGNNYWLIKNSWGEE  LDHGLLLVGYGY----------------EGHESENRKYWLLKNSHGER  LDHGVLVVGFG-------------------TDESGEDYWLVKNSWGTT  LNHGLVLLGFG-------------------TDEQGNRYWICQNSFSQR  GGHAVKLMGWGE--------------------EEGVPYWLCANSWNTD  GGHAIKLMGWGE--------------------EDGVPYWLCANSWNTD  GGHAVRLLGWGE--------------------ENNVPYWLIANSWNSD  GGHAVRLLGWGE--------------------ENNVPYWLIANSWNTD  GGHAVRLLGWGE--------------------ENGVPYWLIANSWNSD  GGHAIRILGWGV--------------------ENGTPYWLVANSWNTD  GGHAIRILGWGV--------------------ENGVPYWLAANSWNLD  GGHAVKILGWGE--------------------ENGVPYWLCANSWNTD  GGHAIRILGWGVWG------------------EEKIPYWLIGNSWNTD  GGHAIRIIGWGV--------------------ENKTPYWLIANSWNED  GGHAIRIIGWGV--------------------EKRTPYWLIANSWNED  GGHAVKLIGWGI--------------------DDGIPYWTVANSWNTD  GGHAVKIIGWGV--------------------ENGVDYWLIANSWGTS  GGHAIKIIGWGT--------------------ESGSPYWLVANSWGVN  GGHAVKILGWGV--------------------DNGTPYWLVANSWNVA  INHVVSVAGWGIS--------------------DGTEYWIVRNSWGEP  INHIISVAGWGVSN-------------------DGIEYWIVRNSWGEP  IDHIISVHGWGVDH------------------ESGVEYWIGRNSWGEP |
| --- | --- | --- |

Figure S2: S01 family catalytic motif (active site shown in blue)

| Human_CFAD[P00746]  Mouse_CFAD[P03953]  Human_GRAM[P51124]  Human_CFD[NP_999875]  Human_GRAK[P49863]  Mouse_granzymeK[O35205] Human_GRAA[P12544]  Human_CATG[P08311]  Mouse_CATG[P28293]  Human_GRAH[P20718]  Human_CELA2[P08246]  Mouse_ELNE[Q3UP87]  Human_CTRB[Q6GPI1]  Mouse_CTRA1[NP_075671]  Human_CTRC[Q99895]  S.mansoni_C4Q5F5[Smp_030350]  human_TM11E[Q9UL52]  Human_HEPS[P05981]  Fruit_fly_TRYA[P04814]  Fruit_fly_TRYDG[P42276]  Fruit_fly_TRYB[P35004]  Fruit_fly_TRYE[P35005]  Fruit_fly_TRYT[P42278]  Fruit_fly_TRYU[P42279]  Fruit_fly_TRYZ[P42280]  Human_BSSP4[Q9GZN4]  Mouse_BSSP4[Q9ER10]  Mouse_PRS27[Q8BJR6]  Human_PRS33[Q8NF86]  Mouse-TRY6[NP_001074868]  C.elegans_TRY1[NP_494910]  Human_TRYB1[P15157]  Human_TRYD[Q9BZJ3]  Human_PRS42[Q7Z5A4]  Mouse_PRS44[Q402U7]  Human_TRY2[P07478]  Human_TRY6[Q8NHM4]  Human_TRY3[P35030]  human_TRY1[P07477]  Mouse_TRY9[Q9QUK9]  Mouse_TRY8[Q9R0T7]  Mouse_TRY16[Q9Z1R9]  Mouse-TRY10[NP_001034085]  Mouse_TRY1[NP_075822]  Mouse_TRY5[EDL13560]  Mouse_TRY4[Q9CPN7]  Human_PRS58[Q8IYP2]  Human_KLK5[Q9Y337]  Mouse_KLK4[Q9Z0M1]  Human_KLK2[P20151]  Human_KLK15[Q9H2R5]  Mouse_KLK11[Q9QYN3]  Mouse_KLK13[Q8CGR6]  Human_KLK14[Q9P0G3]  Human_KLK12[NP_062544]  Human_KLK8[NP_653088]  Mouse_KLK6[EDL22684]  Human_FA9[P00740]  Mouse_FA10[O88947]  Human_FA7[P08709]  Human_CFAI[P05156]  S_mansoni_CERCP12546  S_mansoni_G4M0X8[Smp_119130]  TSO_Scaffold00011.gene1492  TSO_Scaffold00036.gene3378  C_elegans_TRY3[NP_500999]  Human_C1RL[Q9NZP8]  TSO_LongOrf.asmbl_11010  TSO_Scaffold00063.gene4723  TSO_Scaffold00158.gene7407  Human_CFAB[P00751]  C_elegans_TRY5[CCD68623]  C_elegans_TRY4[NP_508030]  S_japonicum_C1LF14  C_elegans_TRY10[CBW48359] | LVAEQWVLSAAHCLEDAADG-KV  LLDEQWVLSAAHCMDGVTDDDSV  LVHPKWVLTAAHCL-AQR-MAQL  LLRARWVVSAAHCF-SHRDLRTG  LIDPQWVLTAAHCQYRFTKGQSP  LIHPQWVLTAAHCYSWFPRGHSP  LIAKDWVLTAAHCN----LNKRS  LVREDFVLTAAHCW-----GSNI  LVREDFVLTAAHCL-----GSSI  LVRKDFVLTAAHCQ-----GSSI  LIAPNFVMSAAHCVAN-VNVRAV  LIARNFVMSAVHCVNG-LNFRSV  LISEDWVVTAAHCG----VRTS-  LISPNWVVTAAHCQ----VTPGR  LIASNFVLTAAHCI----SNTRT  LISAQWVMTAAHCIQP-MPDPKR  LINATWLVSAAHCFTT-YKNPAR  LLSGDWVLTAAHCFPERNRVLSR  IYSANIIVTAAHCLQS--VSASV  IYSSNVIVTAAHCLQS--VSASV  IYSARVIVTAAHCLQS--VSASS  IYSHDIVITAAHCLQS--IEAKD  LINEDTVVTAAHCLVG--RKVSK  ILDAVTIATAAHCVYN--REAEN  IFNETTIVTAAHCVIG--TVASQ  LLTSRWVITAAHCFKDNLNKPYL  LLTNRWVVTAAHCFKSNMDKPSL  LIAPTWVLTAAHCFS-NTSDISI  LIAPQWVLTAAHCFP-RRALPAE  LIAPQWVLTAGHCFP-RRVWPSE  LIDPNFVLTAAHCFA-KDRRPTS  LIHPQWVLTAAHCLGPDVKDLAT  LIHPQWVLTAAHCVEPDIKDLAA  LVTATWVLTAGHCISSRFH----  LISKWWVITAAHCVYGHLD----  LISEQWVVSAGHCYKSRIQVR--  LISEQWVVSAGHCYKPHIQVR--  LISEQWVVSAAHCYKTRIQVR--  LINEQWVVSAGHCYKSRIQVR--  LINDQWVVSAAHCYKTRIQVR--  LINDQWVVSAAHCYKSRIQVR--  LINDQWVVSAAHCYKTRIQVR--  LINDQWVVSAAHCYKSRIQVR--  LINSQWVVSAAHCYKSRIQVR--  LISDQWVLSAAHCYKRRLQVR--  LISDQWVLSAAHCYKRRLQVR--  LIHPLWVITAAHCNLPKLRVI--  LVHPQWLLTAAHCRKKVFRVR--  LVHPQWVLSAAHCLQESYIVG--  LVHPQWVLTAAHCLKKNSQVW--  LISPHWVLSAAHCQSRFMRVR--  LIAPKWLLTAAHCRKPHYVIL--  LVHPKWVLTAAHCRKDGYTVH--  LLSGQWVITAAHCGRPILQVA--  LIDHRWVLTAAHCSGSRYWVR--  LVGGNWVLTAAHCKKPKYTVR--  LIDPQWVLTAAHCKKPNLQVI--  IVNEKWIVTAAHCVETGVKIT--  ILNEFYILTAAHCLHQARRFK--  LINTIWVVSAAHCFDKIKNWRNL  YIGGCWILTAAHCLRASKTHR-Y  LVSTRAVLTAGHCVCSPLPVIRV  LVSTRAVLTAGHCVCSPLPVIRV  LIAPRWILTAAHCMFAYTDDGKQ  LISASWILTAKHCFDSQLDPSLT  VIDDFWLVTAAHCALQLQTRS--  LLGDRWILTAAHTVYPKDSVSLR  LIAPSWLITAAHCLSELVPDKVL  LIAPQWVLTAAHCLKPIFGSSAA  LVSPRHLITAAHCVTKYNSTTAL  VVSEYFVLTAAHCFTVDDKEHSI  LITLKHVLTAAHCFQKHFGAKKE  IISPYHIITAAHGFITTIGSRGN  IISSNKILTAGHCACGDPTYEVH  LIAPSIVITSAHCVFSGDDFAVT | IDHDLLLLQLSEKATLG-  LEDDLILFKLSQNASLG-  LENDLALLQLDGKVKPS-  HANDICLLRLNGSAVLG-  QSNDIMLVKLQTAAKLN-  ASHDIMLIKLRTAAELN-  REGDLKLLQLTEKAKIN-  IQNDIMLLQLSRRVRRN-  IRNDIMLLQLRRRARRS-  FSNDIMLLQLERKAKWT-  LLNDIVILQLNGSATIN-  LLNDIVIIQLNGSATIN-  VNNDITLLKLATPARFS-  MNNDLTLLKLASPARYT-  LRNDIALIKLAEHVELS-  YANDIALLRLQTPANLDN  HDYDISLAELSSPVPYT-  NSNDIALVHLSSPLPLT-  MVNDIAVIRLSSSLSFS-  MVNDIAIIKINGALTFS-  MVNDIAVLHLSSSLSFS-  MVNDIAIIRIESDLSFR-  MEYDVGILKLDEKVKET-  MDNDIALVVVDPPLPLD-  YNNDIAILFVDPPLPLN-  ACADIALVRLERSIQFS-  THADIALVRLEHSIQFS-  SSADVALVELQGPVTFT-  ARGDLALLQLRRPVPLS-  ARGDLALLQLRHPVSLS-  SSYDFAIMRIHPPVNTS-  G-ADIALLELEEPVNIS-  G-ADIALLELEEPVNIS-  IRNDLALLQLQHPVNFT-  VVHDIALVLLAFPVNYS-  LDNDILLIKLSSPAVIN-  LNNDIMLIKLSTPAVIN-  LDNDIMLIKLSSPAVIN-  LNNDIMLIKLSSRAVIN-  LNNDIMLIKLASPVTLN-  LNNDIMLIKLASPVTLN-  LNNDIMLIKLSSPVTLN-  LDNDIMLIKLSSPVTLN-  YNNDIMLIKLKTAATLN-  VDNDIMLIKLKSPAILN-  VDNDIMLIKLKSPAILN-  IDHDIMLIKLKTEAELN-  HSNDLMLIKLNRRIRPT-  FANDLMLIKLNESVIES-  SSHDLMLLRLSEPAKIT-  HRNDIMLLRLVQPARLN-  HRNDIMLVKMSSPVFFT-  HDHDIMLLELKSPVQLS-  HDNDLMLLQLQQPARIG-  HEHDLRLLRLRLPVRVT-  HNHDLMLLQLRDQASLG-  HDNDIMMVHLKNPVKFS-  YNHDIALLELDEPLVLN-  YDYDIAVLRLKTPITFR-  TNHDIALLRLHQPVVLT-  YQNDIALIEMKKDGNKK-  SGFDIAIVMLAQMVNLQ-  SGFDIAIVMLAQMVNLQ-  LENDIALLKLKEHVLVHR  VLDDIALLKLKLPVAFN-  ADNDIALLRISSDLSKLG  FSGDIALLELQHSIPLG-  QGFDVALLKMDEPVEFG-  SPFDVALLRLETPVDIVL  LVHDMAILKLKKPVAYS-  YDYDVALIKLKNKLKYG-  QGNDIVILELESTIDDVE  KGHDWAIVEVEKRIHFS-  GSPDISILTVNRPFNIVK  ANDDVAVIFLPQRADVCH | ESNRRDSCKGDSGGPLVCGGV  ESNRRDTCRGDSGSPLVCGDA  DSKDQAPCKGDSGGPLVCGKG  DSHRRGFCSADSGGPLVCRNR  AKGQKDSCKGDSGGPLICKGV  ARGQKDSCKGDSGGPLICKGI  LRGGRDSCNGDSGSPLLCEGV  RRERKAAFKGDSGGPLLCNNV  PRERKSAFRGDSGGPLVCSNV  PKKTQTGFKGDSGGPLVCKDV  RGRQAGVCFGDSGSPLVCNGL  PRRQAGICFGDSGGPLVCNNL  ASG-VSSCMGDSGGPLVCQK-  GSG-ASSCQGDSGGPLVCQK-  GDGVISACNGDSGGPLNCQLE  KQGGIDACQGDSGGPLLCYVG  LEGKTDACQGDSGGPLVSSDA  PEGGIDACQGDSGGPFVCEDS  -ASGKDACQGDSGGPLVSGG-  -ASGKDACQGDSGGPLVSGG-  -ASGKDSCQGDSGGPLVSGG-  -APHKDACQGDSGGPLVSGD-  -EKKKDACQGDSGGPLAVGN-  SEGGKDACQGDSGGPLVVAN-  GVGGADACQGDSGGPLAVRD-  LEGERDACLGDSGGPLMCQVD  LEGERDACLGDSGGPLMCQVD  AEGKKDACKGDSGGPLVCLVD  PQGHKDACQGDSGGPLTCLQS  RRGHKDACQGDSGGPLTCMES  SYGKIDSCQGDSGGPLMCARD  TR--RDSCKGDSGGPLVCKVN  EN--HDSCQGDSGGPLVCKVN  EQG-KDSCQGDSGGRLACEYN  EKG-GDACQGDSGGPLVCEFN  LEGGKDSCQGDSGGPVVSNGE  LEGGKDSCQGDSGGPVVCNGQ  LEGGKDSWKRDSGGPVVCNGQ  LEGGKDSCQGDSGGPVVCNGQ  LEGGKDSCQGDSGGPVVCNGQ  LEGGKDSCQGDSGGPVVCNGQ  LEGGKDSCQGDSGGPVVCNGE  LEGGKDSCQGDSGGPVVCNGQ  LEGGKDSCQGDSGGPVVCNGQ  LEGGKDSCDGDSGGPVVCNGE  LEGGKDSCDGDSGGPVVCNGE  VPGRRQPCKEVSAAPAICNGM  DKAGRDSCQGDSGGPVVCNGS  GQDQKDSCNGDSGGPIVCNRS  WTGGKDTCGGDSGGPLVCNGV  EGRGAESCEGDSGGPLVCGGI  RKEGKDSCQGDSGGPLVCNGS  KEGGKDSCEGDSGGPLICNGK  PQGGKDSCQGDSGGPLVCRGQ  VPG-QDACQGDSGGPLVCGGV  SKG-ADTCQGDSGGPLVCDGA  MKEGNDSCQGDSGGPLVCGGR  HEGGRDSCQGDSGGPHVTEVE  EAKLEDACQGDSGGPHVTRFK  SDGSKDSCKGDSGGPHATHYR  YDGSIDACKGDSGGPLVCMDA  QNFGQLPAPGDSGGPLLPSLQ  QNFGQLPAPGDSGGPLLPSPQ  FKRNIGICSGDSGGPLVCDFE  NSESTDACQYDSGGPMMCRSG  -AYLHGTAPGDSGGPLLIHKS  ETQRHSVCQGDSGSVYVVWDN  -EKGKDTCRGDSGGGLFCQNX  -SPNKGVCAGDTGGGLFCRNE  -EENKKVWLGDSGGGLYCRLR  PYADPNTCRGDSGGPLIVHKR  -EEDKNVCSGDSGGGLTFHQS  NYSAPRTCHGDSGGGLEYRDD  DMNNQGPDVGDSGGPIFNENG  VTGSSRACMGDSGSPVYCFVN |
| --- | --- | --- | --- |
